# Ocular manifestations of renal ciliopathies

**DOI:** 10.1007/s00467-023-06096-5

**Published:** 2023-08-30

**Authors:** Omar Salehi, Heather Mack, Deb Colville, Debbie Lewis, Judy Savige

**Affiliations:** 1 Department of Medicine (Melbourne Health and Northern Health), The University of Melbourne, Royal Melbourne Hospital, Parkville, VIC 3050 Australia; 2 University Department of Surgery (Ophthalmology), Royal Victorian Eye and Ear Hospital, East Melbourne, VIC 3002 Australia; 3 Nephrology Department, The Children’s Hospital at Westmead, Westmead, NSW 2145 Australia

**Keywords:** Nephronophthisis, Renal ciliopathy, Coloboma, Inherited retinal degeneration, Oculomotor disorder

## Abstract

Renal ciliopathies are a common cause of kidney failure in children and adults, and this study reviewed their ocular associations. Genes affected in renal ciliopathies were identified from the Genomics England Panels. Ocular associations were identified from Medline and OMIM, and the genes additionally examined for expression in the human retina (https://www.proteinatlas.org/humanproteome/tissue) and for an ocular phenotype in mouse models (http://www.informatics.jax.org/). Eighty-two of the 86 pediatric-onset renal ciliopathies (95%) have an ocular phenotype, including inherited retinal degeneration, oculomotor disorders, and coloboma. Diseases associated with pathogenic variants in *ANK6, MAPKBP1, NEK8*, and *TCTN1* have no reported ocular manifestations, as well as low retinal expression and no ocular features in mouse models. Ocular abnormalities are not associated with the most common adult-onset "cystic" kidney diseases, namely, autosomal dominant (AD) polycystic kidney disease and the AD tubulointerstitial kidney diseases (ADTKD). However, other kidney syndromes with cysts have ocular features including papillorenal syndrome (optic disc dysplasia), Hereditary Angiopathy Nephropathy, Aneurysms and muscle Cramps (HANAC) (tortuous retinal vessels), tuberous sclerosis (retinal hamartomas), von Hippel-Lindau syndrome (retinal hemangiomas), and Alport syndrome (lenticonus, fleck retinopathy). Ocular abnormalities are associated with many pediatric-onset renal ciliopathies but are uncommon in adult-onset cystic kidney disease. However the demonstration of ocular manifestations may be helpful diagnostically and the features may require monitoring or treatment.

**Supplementary Information:**

The online version contains supplementary material available at 10.1007/s00467-023-06096-5.

## Introduction

Renal ciliopathies account for at least 15% of children and 5% of adults with kidney failure [1–4]. It is important to identify these diseases because treatments that slow disease progression are increasingly available and because other organs may also be affected [5].

Renal ciliopathies result from pathogenic variants in more than 100 genes (https://panelapp.genomicsengland.co.uk/). The cysts arise mainly from abnormal cilium signaling in the tubules and collecting ducts [2]. The primary cilia on the surface of the tubular epithelial cells function as antennae that sense mechanical, osmotic, and chemical stimuli and signal through pathways that are responsible for maintaining normal tubular structure. These pathways control cell proliferation, differentiation, and orientation, with disruption resulting in cyst development.

The clinical features of renal ciliopathies manifest in organs where the affected genes are expressed and ciliary signaling is important, such as the liver, heart, lungs, skeleton, nerves, and eyes [6–8]. The pattern of extra-renal involvement may be helpful diagnostically.

Renal ciliopathies in children have mainly autosomal recessive (AR) inheritance and include the various forms of nephronophthisis, as well as Senior-Loken, Joubert, Bardet-Biedl, Meckel, and Jeune syndromes, cranioectodermal dysplasia, and the orofaciodigital syndromes (Table 1). These are often associated with ocular abnormalities.

**Table 1:** Renal ciliopathy syndromes with a mainly pediatric presentation and their ocular associations

Disease	Mode of inheritance, population frequency	Age at presentation	Kidney features	Characteristic ocular and extra-renal features
Nephronophthisis [4]	AR; one in 50–100,000	Infantile, juvenile, adolescent	Loss of cortico-medullary differentiation, small cysts, kidney failure	Inherited retinal degeneration, very variable extra-renal features including neurological, skeletal, hepatic and respiratory abnormalities in up to 20%
Senior-Loken syndrome	AR; one in one million [9]	Congenital to adolescence	Nephronophthisis, kidney failure	Leber congenital amaurosis
Joubert syndrome [10]	AR; one in 100,000	Infancy	Nephronophthisis, cystic kidneys, kidney failure	Inherited retinal degeneration; vertical nystagmus; cerebellar vermis hypoplasia (molar tooth sign on MRI), developmental delay, hypotonia
Bardet-Biedl syndrome [11]	AR; one in 14–100,000	Infancy	Nephronophthisis, kidney failure	Inherited retinal degeneration; obesity, polydactyly, abnormal genitalia, intellectual disability
Meckel syndrome [12]	AR; one in 135,000	Embryonic lethal	Renal cystic dysplasia	Occipital encephalocele and polydactyly; retinal degeneration, cataract, corneal dysgenesis
Jeune syndrome [13]	AR; unknown	Infancy	Cystic kidneys, kidney failure	Short ribs, short limbs, polydactyly
Cranioectodermal dysplasia [14]	AR; < 50 cases worldwide	Infancy	Nephronophthisis, kidney failure	Retinal degeneration (one report); intellectual disability; skeletal (narrow thorax, short limbs, brachydactyly, dental/skin/hair abnormalities, facial dysmorphia
Orofaciodigital syndrome [15]	XL; one in 50–250,000	Infancy	Fused or polycystic kidneys, proteinuria	Hypertelorism; retinal degeneration,; intellectual disability; tongue/dental abnormalities, cleft lip/palate, facial dysmorphia, digital malformation

The most common adult-onset diseases included here are autosomal dominant (AD) polycystic kidney disease (ADPKD) and AD tubulointerstitial kidney disease (ADTKD), previously known as “medullary cystic kidney disease”) due to pathogenic variants in *MUC1, UMOD, REN*, or *HNF1B. *ADTKD is characterised by microcysts rather than cysts. Tuberous sclerosis is common but results less often in kidney failure. Other diseases associated with kidney cysts include ARPKD, the rare von Hippel-Lindau syndrome, papillorenal syndrome, and hereditary angiopathy, nephropathy, aneurysms, and muscle cramps or HANAC. X-linked (XL) and AD Alport syndrome are also associated with kidney cysts, but their pathogenesis is unclear [16, 17]. In addition, acquired cystic kidneys are common in individuals undergoing dialysis [18]. Cystic liver disease without significant kidney cysts usually results from variants in other genes [19].

The eye is often involved in genetic kidney disease because, despite their different functions, the eye and kidney share developmental pathways and some specialized structural features [20]. Both the kidney and the eye undergo organogenesis in the 4th to 7th embryonic week, and perturbations during this period result in kidney and ocular phenotypes [21]. Primary cilia on the surface of the tubule epithelium are critical in maintaining tubular structure and orientation, and disruption leads to cyst formation. Cilia in the photoreceptor outer segment relay sensory stimuli to the brain via the visual pathway, and defects result in retinal degeneration [22]. The retinal pigment cells—Bruch’s membrane—choriocapillaris and glomerular filtration barrier also share structural features [23].

While genetic testing has become more widely available, ocular phenotyping is a simple, non-invasive method that may suggest the diagnosis. Genetic testing also indicates the mode of inheritance and other at-risk family members. In addition, ophthalmic examination identifies vision-threatening conditions that require regular monitoring and, in some cases, treatment.

The aim of this study was to review the reported ophthalmic associations of renal ciliopathies in children and cystic diseases in adults in order to identify features that might help the nephrologist recognize the disease’s genetic basis. The study also examined whether individual genes were expressed in the retina or associated with ocular features in mouse models, which suggested ocular abnormalities might yet be found in the corresponding human disease.

## Strategy

The major genes affected in the renal ciliopathies and other forms of cystic kidney disease were identified from the Genomics England Renal ciliopathy panel (v1.64; https://panelapp.genomicsengland.co.uk/) and from reviews [16, 24]. Gene names were then searched in OVID Medline together with (“AND”) the following search terms: “ocular,” “ophthal*,” “optic*,” “eye,” “vision*,” “cornea,” “iris”, “pupil,” “lens,” “retina,” “choroid,” “fovea,” “macula,” and “optic nerve” to identify relevant manuscripts. Abstracts were reviewed, and relevant manuscripts examined for ocular abnormalities. Inclusion criteria were studies describing a renal ciliopathy or cystic kidney disease, associated ocular phenotypes, and publication in a peer-reviewed journal. Articles were excluded if they were not written in English or if the full text was not available online. The search was conducted between August and September 2020 and reviewed in August–October 2022. Gene names and corresponding diseases were also searched for “eye” features in OMIM in October 2022.

Expression of the genes was examined in the retina in the Human Protein Atlas (https://www.proteinatlas.org/humanproteome/tissue); and the effects of the corresponding genes on ocular manifestations examined in mouse models in the Mouse Genome Informatics website (http://www.informatics.jax.org/) in October 2022.

## Renal ciliopathies with a mainly pediatric presentation

Eighty-six genes associated with a mainly pediatric presentation were examined from the renal ciliopathy green and amber lists after *PKD1, 2, PKHD1, MUC1, UMOD*, and *HNF1B* had been excluded (Table 2).

**Table 2:** Ocular phenotypes, expression, and mouse ocular features associated with pediatric renal ciliopathies

Gene (OMIM)	Disease (s)	Ocular phenotype (OMIM and literature)	Retinal expression*	Mouse ocular phenotype**
*AHI1 (*608894)	Joubert syndrome 3 (608629)	Coloboma, inherited retinal degeneration oculomotor apraxia, nystagmus, strabismus, ptosis, epicanthal folds (OMIM)	27.2 TPM	Retinal Degeneration
*ALMS1 *(606844)	Alstrom syndrome (203800)	Cone-rod dystrophy; pigmentary retinopathy; nystagmus; cataracts; optic neuropathy; hyperopia; constricted visual field (OMIM)	10.4 TPM	Retinal degeneration
*ANK6 *(615370)	Nephronophthisis 16 (615382)	No ocular abnormalities reported in OMIM nor in search	2.2 TPM	None noted
*ARL13B *(608922)	Joubert syndrome 8 (612291)	Pigmentary retinopathy, optic disc pallor, abnormal eye movements (OMIM)	18.2 TPM	Retinal degeneration
*ARL6 *(608845)	Bardet-Biedl syndrome 3 (600151)	Inherited retinal degeneration (OMIM)	46.3 TPM	Retinal degeneration
*ARMC9 *(617612)	Joubert syndrome 30 (617622)	Inherited retinal degeneration, abnormal eye movements, ptosis (OMIM)	71.5 TPM	Retinal degeneration
*B9D2 *(611951)	Meckel syndrome.10, Joubert syndrome 34 (614175)	Ptosis, epicanthus, small palpebral fissures (OMIM); abnormal eye movements	1.2 TPM	Abnormal optic cup, eye muscle morphology; aphakia
*BBS1 *(209901)	Bardet-Biedl syndrome 1 (209900)	Rod-cone dystrophy, strabismus, cataracts (OMIM); Inherited retinal degeneration. For all Bardet-Biedl syndrome subtypes	21.4 TPM	Retinal degeneration, anophthalmia
*BBS10* (610148)	Bardet-Biedl syndrome 10 (615987)	Inherited retinal degeneration	11.4 TPM	Retinal degeneration
*BBS12 *(610683)	Bardet-Biedl syndrome 12 (615989)	Inherited retinal degeneration	17.6 TPM	Retinal degeneration
*BBS2 *(606151)	Bardet-Biedl syndrome 2 (615981)	Inherited retinal degeneration	86.5 TPM	Retinal degeneration
*BBS4* (600374)	Bardet-Biedl syndrome 4 (615982)	Inherited retinal degeneration	45.4 TPM	Retinal degeneration, optic nerve atrophy
*BBS5 *(603650)	Bardet-Biedl syndrome 5 (615983)	Inherited retinal degeneration	11.3 TPM	Retinal degeneration
*BBS7 *(607590)	Bardet-Biedl syndrome 7 (615984)	Inherited retinal degeneration	56.6 TPM	Retinal degeneration, abnormal lens
*BBS9 *(607968)	Bardet-Biedl syndrome 9 (615986)	Inherited retinal degeneration, cataract, optic nerve dysplasia, nystagmus	21.9 TPM	None noted
*BBIP1 *(613605)	Bardet-Biedl syndrome 18 (615995)	Inherited retinal degeneration, cataracts	50.1 TPM	Retinal degeneration
*C5orf42 *(614571)	Joubert syndrome 17 (614615); orofaciodigital syndrome VI (277170)	Oculomotor apraxia (OMIM); hypertelorism, epicanthal folds, nystagmus, oculomotor apraxia	5.7 TPM	Abnormal eye morphology
*CC2D2A *(612013)	Joubert 9 (612285), Meckel syndrome 6 (612284), COACH syndrome 2 (619111)	Astigmatism, coloboma, inherited retinal degeneration, oculomotor apraxia, nystagmus, cataract (OMIM)	84.7 TPM	Anophthalmia, microphthalmia, retinal degeneration
*CENPF *(600236)	Stromme syndrome (243605)	Microphthalmia, microcornea, anterior chamber defects, iris coloboma, optic nerve hypoplasia, cataracts, hypertelorism, tortuous retinal vessels	1.0 TPM	None noted
*CEP104* (616690)	Joubert syndrome 25 (616781)	Oculomotor apraxia (OMIM), nystagmus, inherited retinal degeneration	9.0 TPM	None noted
*CEP164* (614848)	Nephronophthisis 15 (614845)	Inherited retinal degeneration; Leber congenital amaurosis, nystagmus (OMIM)	26 TPM	None noted
*CEP290* (610142)	Nephronophthisis 6, Joubert 5 (610188), Bardet-Biedl 14 (615991), Meckel syndrome 4 (611134)	Inherited retinal degeneration, coat-like exudative vasculopathy; congenital amaurosis, nystagmus, retinal coloboma; oculomotor apraxia (OMIM)	7.2 TPM	Retinal degeneration
*CEP41* (610523)	Joubert syndrome 15 (614464)	Chorioretinal coloboma, inherited retinal degeneration, oculomotor apraxia (OMIM)	7.7 TPM	None noted
*CEP83* (615847)	Nephronophthisis 18 (615862)	Inherited retinal degeneration, strabismus	5.3 TPM	None noted
*CRB2* (609720)	Ventriculomegaly with cystic kidney disease (219730)	Inherited retinal degeneration	8.1 TPM; high in limiting membrane	Inherited retinal degeneration
*CSPP1* (611654)	Joubert syndrome 21 (615636)	Inherited retinal degeneration oculomotor apraxia, strabismus, ptosis, fused eyes, anophthalmia (OMIM); nystagmus, corneal clouding (rare), cataracts (rare)	2.1 TPM	None noted
*DDX59* (615464)	Oral-Facial-digital syndrome V (174300)	Epicanthus, hypertelorism, telecanthus (OMIM), coloboma, ptosis	17.4 TPM	None noted
*DHCR7* (602858)	Smith-Lemli-Opitz syndrome (270400)	Ptosis, epicanthal folds, cataracts, hypertelorism, strabismus (OMIM); optic atrophy, blepharoptosis, optic nerve hypoplasia	8.7 TPM	Microphthalmia
*DYNC2H1 *(603297)	Jeune syndrome 3 (613091)	Inherited retinal degeneration	10.5 TPM	Abnormal eye morphology
*HYLS1* (610693)	Hydrolethalus syndrome (236680)	Micropthalmia (OMIM); optic nerve coloboma and hypoplasia	4.3 TPM	None noted
*ICK* (612325)	Endocrine-cerebro-osteodysplasia (612651)	Small sunken eyes, fused eye lids (OMIM)	NA	None noted
*IFT122* (606045)	Cranioectodermal dysplasia 1 (218330)	Hypertelorism, epicanthal folds, myopia, nystagmus, inherited retinal degeneration (OMIM)	11.2 TPM	Abnormal eye morphology
*IFT43* (614068)	Cranioectodermal dysplasia 3 (614099), Jeune syndrome 18 (617866)	Inherited retinal degeneration	25.1 TPM	None noted
*INPP5E* (613037)	Joubert syndrome 1 (613037)	Inherited retinal degeneration; abnormal jerky eye movements, oculomotor apraxia, coloboma of the optic nerve; chorioretinal coloboma, epicanthal folds, ptosis (OMIM)	7.4 TPM	Inherited retinal degeneration; abnormal eye morphology; microphthalmia
*INVS* (243305)	Nephronophthisis 2 (602088)	Inherited retinal degeneration	11.8 TPM	None noted
*IQCB1* (609237)	Senior-Loken syndrome 5 (609254)	Leber congenital amaurosis; inherited retinal degeneration (OMIM)	18.9 TPM; medium in photo-receptor cells	Increased corneal thickness
*KIAA0586* (610178)	Joubert syndrome 23 (616490)	Inherited retinal degeneration, nystagmus; abnormal eye movements, coloboma (OMIM)	7.9 TPM	None noted
*KIAA0753* (617112)	Joubert syndrome 38 (619476)	Inherited retinal degeneration, nystagmus, abnormal eye movements, epicanthal folds, oculomotor apraxia (OMIM)	10.8 TPM	None noted
*KIF7* (611254)	Joubert syndrome 7 (200990)	Strabismus, hypertelorism, epicanthal folds, optic atrophy, inherited retinal degeneration, nystagmus, coloboma (OMIM)	0.2 TPM	Microphthalmia, anophthalmia
*LZTFL1* (606568)	Bardet-Biedl syndrome 17 (615994)	Inherited retinal degeneration (OMIM)	15.8 TPM	Retinal degeneration
*MAPKBP1 *(616786)	Nephronophthisis 20 (617271)	No ocular features reported nor in OMIM	9.0 TPM	None noted
*MKKS* (604896)	Bardet-Biedl syndrome (605231); McKusick-Kaufman syndrome (236700)	Inherited retinal degeneration	22.8 TPM	Retinal degeneration
*MKS1* (609883)	Meckel 1 (249000); Bardet-Biedl 13 (615990); Joubert syndrome 28 (617121)	Oculomotor apraxia, nystagmus, microphthalmia, inherited retinal degeneration, coloboma, optic disc pallor, ptosis	6.1 TPM	Anophthalmia; microphthalmia, abnormal eye morphology
*NEK8* (609799)	Nephronophthisis 9 (613824); renal-hepatic pancreatic dysplasia 2 (615415)	No ocular features reported nor in OMIM	0.6 TPM	None noted
*NPHP1* (607100)	Nephronophthisis 1 (256100), Joubert syndrome 4 (609583), Senior-Loken syndrome 1 (266900)	Inherited retinal degeneration, Stargardt-like retinopathy, oculomotor apraxia	10.8 TPM	Retinal degeneration
*NPHP3* (608002)	Nephronophthisis 3 (604387), Meckel syndrome 7 (267010)	Inherited retinal degeneration, cataract, nystagmus	3.1 TPM	None noted
*NPHP4* (607215)	Nephronophthisis 4 (606966), Senior-Loken syndrome (606966)	Coloboma, inherited retinal degeneration, oculomotor apraxia; amblyopia, rotary nystagmus (OMIM)	2.4 TPM	Retinal degeneration
*OFD-1* (300170)	Orofaciodigital syndrome 1 (311200)	Inherited retinal degeneration, bilateral idiopathic demyelinating optic neuritis; epicanthal folds (OMIM)	11.2 TPM	None noted
*PMM2* (601785)	Congenital disorder of glycosylation 1a (212065)	Abnormal eye movements, strabismus, nystagmus, inherited retinal degeneration (OMIM)	1.5 TPM	Abnormal eye morphology
*RPGRIP1L* (610937)	Nephronophthisis 8, Joubert 7 (611560), Meckel 5 (611561)	Coloboma, inherited retinal degeneration, oculomotor apraxia; nystagmus, ptosis (OMIM)	2.9 TPM	Anophthalmia; abnormal optic cup; eye muscles
*SDCCAG8 *(613524)	NPHP10, Bardet-Biedl 16 (615993)	Inherited retinal degeneration	3.7 TPM	Retinal degeneration
*TCTN1* (609863)	Joubert syndrome 13 (614173)	No ocular features reported nor in OMIM	3.0 TPM	None reported
*TCTN2* (613846)	Joubert syndrome 24 (616654)	Anophthalmia, nystagmus	17.6 TPM	Anophthalmia; microphthalmia
*TCTN3* (613847)	Joubert 18 (614815); orofaciodigital syndrome IV (258860)	Abnormal eye movements; hypertelorism, epicanthal folds (OMIM)	22.2TPM	Anophthalmia)
*TMEM107 *(616183)	Meckel syndrome 13 (617562); Joubert syndrome (617562), orofaciodigital syndrome XVI (617563)	Inherited retinal degeneration, oculomotor apraxia, ptosis (OMIM)	29.6 TPM	Microphthalmia (MGI)
*TMEM138* (614459)	Joubert syndrome 16 (614465)	Coloboma, inherited retinal degeneration, oculomotor apraxia, nystagmus, strabismus	50.7 TPM	None noted
*TMEM216* (613277)	Joubert 2 (608091), Meckel syndrome 2 (603194)	Coloboma, inherited retinal degeneration, oculomotor apraxia, nystagmus, strabismus	11.8 TPM	None noted
*TMEM231* (614949)	Joubert 20 (614949), Meckel 11 (615397)	Inherited retinal degeneration, oculomotor apraxia	5.2 TPM	Anophthalmia; microphthalmia
*TMEM237* (614423)	Joubert syndrome 14 (614424)	Morning glory anomaly, retinal coloboma, nystagmus, strabismus; hypertelorism, ptosis, epicanthal folds (OMIM)	89.3 TPM	None noted
*TMEM67 *(609884)	Nephronophthisis 11 (613550), Joubert 6 (610688), Meckel 3 (607361)	Ptosis, anisocoria, chorioretinal coloboma, inherited retinal degeneration, oculomotor apraxia, nystagmus	6.1TPM	Retinal degeneration
*TRAF3IP1 *(607380)	Senior-Loken syndrome 9 (616629)	Inherited retinal degeneration, nystagmus, strabismus (OMIM); iris patterns	6.7 TPM	Microphthalmia, thick cornea
*TTC21B* (612014)	NPHP12, (613820) Jeune syndrome 4	Pathological myopia associated with chorioretinal atrophy, choroidal neovascularization and traction retinopathy	7.5 TPM	Shortened primary cilia
*TTC8* (608132)	Bardet-Biedl syndrome 8 (613464)	High myopia, inherited retinal degeneration, optic neuropathy (OMIM)	37.4 TPM	Retinal degeneration
*TXNDC15* (619879)	Meckel syndrome 14 (619879)	Hypertelorism, microphthalmia (OMIM)	38.5 TPM	None noted
*WDPCP* (613580)	Bardet-Biedl syndrome 15 (615992)	Inherited retinal degeneration	12.2 TPM	Anophthalmia
*WDR19* (608151)	NPHP13 (614377), Jeune 5, cranioectodermal dysplasia 4	Inherited retinal degeneration, nystagmus	13.0 TPM	Anophthalmia
*WDR35* (613602)	Cranioectodermal dysplasia 2 (613610), Jeune 7	Optic nerve coloboma, nystagmus, hypermetropia, strabismus, amblyopia	12.6 TPM	None noted
*WDR60* (615462)	Jeune syndrome (615503)	Inherited retinal degeneration (OMIM)	14.1 TPM	None noted

These genes are from the Genomics England Ciliopathy genes (green and amber lists). Ophthalmological features are described as reported in the literature and some terms overlap. *From the Human Protein Atlas (https://www.proteinatlas.org/humanproteome/tissue); and **from the Mouse Genome Informatics website (http://www.informatics.jax.org/)

Eighty-two of these genes (95%) had a reported ocular phenotype in human disease. Four renal ciliopathy genes (*ANK6, MAPKBP1, NEK8, TCTN1)* had no ocular phenotype in human disease and were associated with low retinal expression (< 10 transcripts per million, TPM) and no mouse ocular phenotype.

Eighty-five of the 86 genes (99%) were expressed in the retina. The exception was *ICK* which is associated with endocrine cerebro-osteodysplasia, but its kidney phenotype is unclear. Fifty-eight of these genes (67%) were expressed at > 10 TPM in the retina. Sixty genes (70%) had an ocular phenotype in the corresponding mouse model. Thirteen genes were expressed both at low levels in the retina and not associated with an ocular phenotype in the mouse model, but nine of these had a reported phenotype in human disease.

## Ocular phenotypes in nephronophthisis and other renal ciliopathies

**Fig. 1:**
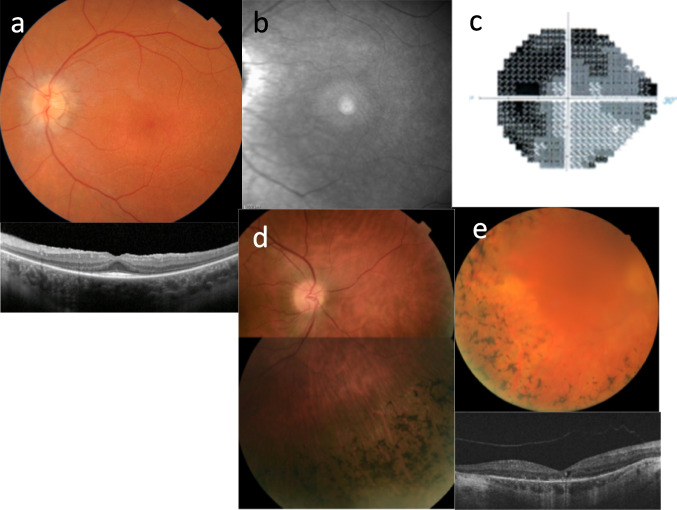
Inherited retinal degeneration in renal ciliopathies demonstrating **a** a waxy optic disc, attenuated arterioles, and widespread mottling of the retinal pigment epithelium in nephronophthisis; with OCT scan demonstrating outer retinal atrophy with a small area of retained subfoveal photoreceptors (below); **b** infrared fundus image demonstrating a ring of retained normal retina; **c** automated visual field test demonstrating constricted visual field; **d** inferonasal retinal pigmentation in an adult with Bardet-Biedl syndrome; and **e** retinal thinning and intraretinal pigment migration with OCT scan demonstrating associated cystoid macular edema (below)

**Fig. 2:**
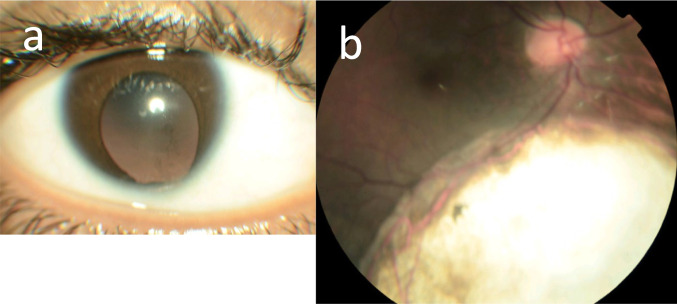
Optic disc anomalies and coloboma in renal ciliopathies demonstrating **a** iris coloboma and **b** large chorioretinal coloboma

The descriptions for the clinical features of these diseases often overlap**,** and almost all of these genes have an ocular phenotype. The most common ocular abnormalities are inherited retinal degeneration, coloboma, and oculomotor abnormalities (Tables 1, 2, 3; Figs. 1, 2).

**Table 3:** Ocular manifestations of the pediatric renal ciliopathies

Ophthalmic feature	Examples of ciliopathies with these ophthalmic features	Examples of genes with pathogenic variants associated with these ophthalmic features
Hypermetropia (long-sightedness)	Cranioectodermal dysplasia; Jeune syndrome	*WDR35*
Myopia (short-sightedness)	ARPKD; Jeune syndrome; nephronophthisis	*TTC21B*
Astigmatism	Joubert syndrome; Meckel syndrome	*CC2D2A*
Congenital size anomaly (anophthalmia, microphthalmia)	Joubert syndrome; Meckel syndrome; Bardet-Biedl syndrome	*MKS1, TCTN2*
Colobomata (iris, optic disc, chorioretinal)	Nephronophthisis; Joubert syndrome; Meckel syndrome; renal coloboma syndrome; tuberous sclerosis complex	*AHI1, CC2D2A, CEP41, NPHP4, TMEM138, MSK1, TMEM216, TMEM237, TSC1, TSC2, WDR35*
Eyelid abnormalities	Nephronophthisis; Joubert syndrome; Meckel syndrome	*TMEM67*
Corneal abnormalities	Joubert syndrome	*CEP41, COL4A5*
Anterior segment malformations	Tuberous sclerosis complex	*TMEM67, TSC1, TSC2*
Lens cataracts	Tuberous sclerosis complex	*TSC1, TSC2, COL4A5*
Optic disc abnormalities	Orofaciodigital syndrome; Joubert syndrome; Renal coloboma syndrome	*TMEM237, OFD-1, PAX2*
Retina—inherited retinal degeneration	Nephronophthisis; Joubert syndrome; Bardet-Biedl syndrome; Jeune syndrome; cranioectodermal dysplasia; Meckel syndrome; orofaciodigital syndrome	*AHI1, ARL6, BBIP1, BBS1, BBS2, BBS4, BBS5, BBS9, BBS10, CC2D2A, CEP164, CEP290, CEP41, CEP83, DYNC2H1, IFT122, IFT43, INPP5E, KIAA0586, KIAA0753, INVS, IQCB1, LZTFL1, MKS1, NPHP1, NPHP3, NPHP4, OFD-1, RPGRIP1L, SDCCAG8, TMEM138, TMEM216, TMEM231, TMEM67, TTC8, WDPCP, WDR19, WDR60*
Retina—other abnormalities	Nephronophthisis; tuberous sclerosis complex; Von Hippel-Lindau disease	*TSC1, TSC2, TTC21B, VHL*
Neuro-ophthalmic (strabismus, pupil disorders, Duane syndrome, nystagmus)	Nephronophthisis; Joubert syndrome; Meckel syndrome; Jeune syndrome; cranioectodermal dysplasia; Bardet-Biedl syndrome	*WDR35, AHI1, CC2D2A, CEP120, CEP41, CEP83,, MKS1, RPGRIP1L, TCTN2, TCTN3, TMEM138, TMEM216, TMEM231, TMEM237, TMEM67, WDR19, WDR35*

Individual features are described more fully in Table 2

Senior-Loken syndrome is diagnosed when there is retinal disease as well as nephronophthisis, which occurs in at least 15% of cases [27]. Many gene variants have been reported [28–30]. Pathogenic variants in *IQCB1 (NPHP5*) are always associated with retinal abnormalities.

Joubert syndrome is characterized by nephronophthisis with neurological disease resulting in hypotonia and developmental delay. The “molar tooth sign” on MRI is pathognomonic. Pathogenic variants affect up to 30 different genes. Variants in the *AHI1, CPLANE1, CC2D2A, CEP290*, and *TMEM67* genes represent 40% of all cases [31–33]. Neuro-ophthalmic abnormalities are common, with retinal disease and coloboma occurring less frequently [34].

Bardet-Biedl syndrome includes nephronophthisis, polydactyly, obesity, diabetes, genital anomalies, and inherited retinal degeneration. More than 20 genes are implicated, most of which are found in the “BBSome,” a protein complex involved in ciliary trafficking [35]. Retinal disease occurs in 90% of cases [36] but coloboma and oculomotor disorders are uncommon.

Meckel syndrome is a severe multi-system illness that is often fatal in the perinatal period. Pathogenic variants in the same genes may also result in milder disease, such as Joubert syndrome, with inherited retinal degeneration, coloboma, microphthalmia, or oculomotor disorders [12, 37].

Jeune syndrome (“asphyxiating thoracic dystrophy”) and cranioectodermal dysplasia (Sensenbrenner syndrome) are rare skeletal dystrophies that occur because of disrupted intraflagellar transport. Nephronophthisis is associated with high myopia, inherited retinal degeneration, and neuro-ophthalmic features [14, 38, 39].

Orofaciodigital syndrome type 1 includes malformations of the face, mouth, and digits and is often associated with kidney cysts, fibrocystic liver disease, and neurological, skeletal, and cardiac anomalies [40]. The less common types 2–13 do not appear to have kidney cysts. Ocular abnormalities are limited to hypertelorism and epicanthal folds [41].

## Common ocular phenotypes in pediatric ciliopathies (Table 3; Figs. 1, 2)

### Inherited retinal degeneration

Inherited retinal degeneration (dystrophy, disease) encompasses a heterogeneous group of diseases characterized by photoreceptor degeneration and impaired vision. It is categorized by the photoreceptor type affected, with disease predominantly affecting the rods, cones, or both. Retinitis pigmentosa is the most common form [42]. Rods are affected early with subsequent progression involving the cones. Night blindness due to rod dysfunction is an early feature. Peripheral visual field deficits may progress to tunnel vision. The retina has thinned vessels, abnormal pigmentation, retinal pigment atrophy, and waxy pallor of the optic disc [43].

The diagnosis of Senior-Loken syndrome (nephronophthisis with inherited retinal degeneration) depends on the use of retinal imaging, fundus autofluorescence, optical coherence tomography, and electroretinography [44]. The clinical outcome is highly variable, even among individuals with the same gene variants [45]. Gene therapy is currently being explored as treatment [42, 46–49], and intravitreal antisense oligonucleotide injections have improved visual acuity and oculomotor stability for at least the *CEP290* variants [50].

Leber congenital amaurosis is a severe form of congenital or early-onset inherited retinal degeneration affecting both rods and cones. Presentation is typically in the first few months of life with impaired vision, nystagmus, sluggish pupillary light reflexes, and increased eye rubbing (the “oculo-digital sign”) that may increase the risk of keratoconus [51, 52]. Fundus imaging may be normal early, but vascular attenuation, optic disc pallor, and abnormal pigmentation occur with time [53]. Pathogenic variants in *CEP290* and *RPGRIP1L* are responsible for 20% and 5% of cases, respectively [54].

### Coloboma

Overall coloboma are normally found in one in 2000–10,000 births, and one or both eyes may be affected [55, 56]. Coloboma are common in genetic kidney disease but also occur as an isolated phenomenon, with CAKUT, some genetic glomerulopathies or tubulopathies and with other genetic diseases that do not affect the kidneys.

Coloboma occur from abnormal closure of the embryonic fissure during the sixth to seventh week of gestation. Coloboma of the iris and ciliary body result from abnormal anterior closure, and coloboma of the optic nerve, retina, and choroid from failed posterior closure [57].

Chorioretinal coloboma are detected on fundus imaging as discrete, white lesions commonly affecting the inferonasal quadrant with overlying pigmentation. Complications include a 30% increased risk of retinal detachment possibly with visual field defect [58], and amblyopia [59], cataract, glaucoma, and increased refractive error [60–62].

Optic nerve coloboma vary in size with the largest resembling the “morning glory” anomaly with enlargement, dysplasia, and funneling of the optic disc, radial orientation of retinal vessels, and peripapillary pigmentation [63].

### Neuro-ophthalmological disorders

These are particularly common in nephronophthisis and related ciliopathies, especially Joubert syndrome. Congenital oculomotor apraxia occurs in 80% of individuals with Joubert syndrome [64] and is characterized by the inability to initiate voluntary eye movements, most often horizontal saccades. In order to compensate, individuals use “head thrusting” to bring an object of interest into their field of vision after head control is established at 6 months of age [65, 66]. Head thrusting tends to diminish after the first year of life, and no specific intervention alters disease progression. Various forms of nystagmus have been described including “pendular” and “seesaw” types [67]. Strabismus occurs in most patients with Joubert syndrome and often requires surgical correction [34].

## Renal ciliopathies and cystic kidney disease with a mainly adult-onset presentation

**Fig. 3:**
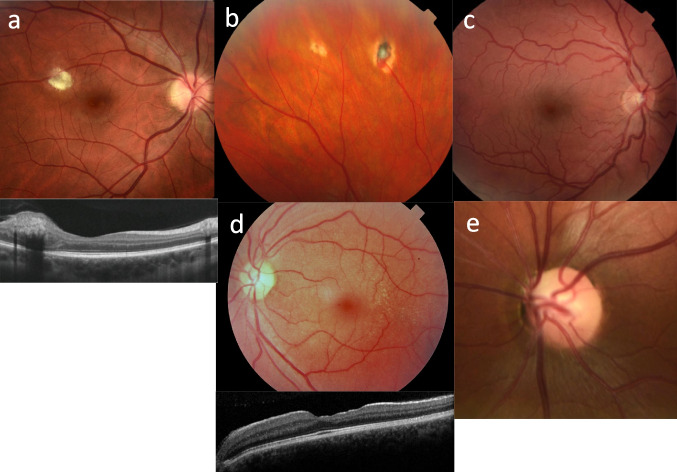
Ocular abnormalities in cystic kidney disease demonstrating **a** tuberous sclerosis with retinal astrocytoma and OCT with intraretinal location (below); **b** von Hippel-Lindau syndrome with treated hemangioma; **c** congenital vascular tortuosity typical of HANAC; **d** X-linked Alport syndrome with fleck dystrophy that spares the macula, and OCT that demonstrates the temporal retinal thinning and characteristic macular profile (below); and **e** renal coloboma syndrome with retinal vessels emerging from the side rather than the center of the optic disc

Ten genes associated with cystic kidney disease that include the commonest genetic causes of cystic kidney disease, ADPKD and ADTKD, were examined (Table 4; Fig. 3). Ocular features are not described in these diseases. In addition, *PKD1* and all ADTKD genes are expressed at very low levels in the human retina. Only *PKD2, GANAB*, and *DNAJB11* are found at levels > 10 transcripts per million (TPM) but still have no reported ocular phenotype in human disease nor in mouse models.

**Table 4:** Overview of genes and clinical features in renal ciliopathies and other cystic diseases with mainly adult presentations

Disease	Gene, mode of inheritance	Population frequency, age at presentation	Kidney features	Characteristic ocular and extra-renal features	mRNA expression in human retina*	Mouse ocular phenotype**
Polycystic kidney disease						
ADPKD (173900) [68]	*PKD1* (601313), AD	1/1,000; 40–70	Bilateral, diffuse large cysts, kidney failure	No ocular features reported consistently (droopy eye lids; reduced corneal cells in individual reports); cysts in liver, pancreas, testis; intracranial aneurysms, cardiac anomalies (mitral valve and aortic root dilatation)	7.4 TPM	None noted
ADPKD (613095)	*PKD2 *(173910)*, AD*	1 in 10,000; 60–80	Bilateral cysts, hypertension	No ocular features reported	14.0 TPM	None noted
PKD 3 (600666)	*GANAB* (104160), AD	Unknown	Kidney cysts, typically mild kidney disease	No ocular features reported; sometimes liver cysts, intracranial aneurysms	79.4 TPM	None noted
PKD 6 with or without polycystic liver disease (618061)	*DNAJB11* (611341), AD	Unknown	Small kidney cysts,	No ocular features reported; sometimes liver cysts, gout	16.1 TPM	None noted
Polycystic kidney disease 5 (617610)	*DZIP1L* (617570), AR	Unknown	Multiple tiny kidney cysts, calcification, onset in childhood, kidney failure	No ocular features reported; no liver cysts or extrarenal features reported	4.6 TPM	None noted
ARPKD (263200)[69]	*PKHD1,* (606702), AR	1/20,000; neonatal	Cysts, fibrosis, kidney failure	No ocular features reported; liver fibrosis and biliary dilation	1.5 TPM	None noted
ADTKD						
ADTKD-*MUC1* (174000) [70]	*MUC1* (158340), AD	Unknown; 30–70	ADTKD—microcysts (tubular dilatation), tubulointerstitial fibrosis, kidney failure	No ocular features reported; gout	1.3 TPM	None noted
ADTKD-*UMOD* (162000)	*UMOD *(191845)*, AD*	Unknown; 30–70	ADTKD	No ocular features reported; gout	0 TPM	None noted
ADTKD-*REN* (613092)	*REN* (179820), AD	Unknown; 30–70	ADTKD	No ocular features reported; hyperuricemia	0.1TPM	None noted
ADTKD-*HNF1B* (137920)	*HNF1B* (189907), AD	Unknown; 30–70	ADTKD	No ocular features reported—coloboma in one case probably coincidental; diabetes, pancreatic atrophy, hypospadias, atrophy of vas deferens	0 TPM	None noted
Other syndromes associated with renal cysts						
Renal-coloboma syndrome (120330) [71]	*PAX2* (167409), AD	Unknown; childhood-adult	Multi cystic dysplastic kidney	Morning glory anomaly, retinal coloboma, pigmentary macular dysplasia, optic nerve cyst, scleral staphyloma	1.2 TPM	Optic disc coloboma; other coloboma, abnormal eye development, abnormal retinal vasculature
Von Hippel-Lindau disease (193300)[72]	*VHL *(608537)*,* AD	1/36,000; adulthood	Cysts, renal cell carcinoma	Hemangioblastoma (retinal, optic nerve), retinal vascular proliferation, neuroendocrine tumors	8.9 TPM	Abnormal retinal vessels
Tuberous sclerosis complex 1 (191100)[73]	*TSC1 *(605284)*,* AD	1/6,000- 10,000; adulthood	Cysts, angiomyolipoma, renal cell carcinoma	Iris hamartoma and hypopigmentation, juvenile cataract (usually anterior subcapsular component), retinal hamartoma, chorioretinal coloboma. skin and neurological anomalies, cardiac rhabdomyoma, pulmonary lymphangioleiomyomatosis	8.3 TPM	Abnormal iris, cornea, eye development, optic cup and posterior eye segments
Tuberous sclerosis Complex 2 (613254)	TSC2 (191092), AD	Adulthood	Cysts, angiomyolipoma, renal cell carcinoma	As for tuberous sclerosis complex 1	17.7 TPM	
HANAC (Hereditary Angiopathy, Nephropathy, Aneurysms, muscle Cramps) (611773)[74]	*COL4A1* (120130), AD	Unknown; adulthood	Hematuria, cysts, kidney failure	Tortuous retinal vessels, retinal hemorrhage, cataracts; cerebral microvascular disease, muscle cramps	1.8 TPM	Cataract, abnormal iris, cornea, abnormal retinal vasculature, microphthalmia
Alport syndrome (301050)[75, 76]	*COL4A5 *(303630)*, XL*	One in 2300; childhood–adulthood	Cysts, hematuria, proteinuria, kidney failure	Anterior lenticonus, cataract; posterior polymorphous corneal dystrophy, fleck retinopathy, temporal macular thinning, giant macular hole; also hearing loss, aortic aneurysms	6.1 TPM	Abnormal lens capsule, and eye physiology

*AD*, autosomal dominant; *AR*, autosomal recessive; *XL*, X-linked; *ADTKD*, AD tubulointerstitial kidney disease; *KF*, kidney failure; *TPM*, transcripts per million; *NA*, not available

^*^Expression in Human Protein Atlas (https://www.proteinatlas.org/humanproteome/tissue); **phenotype in mouse models in Mouse Genomics Initiative (http://www.informatics.jax.org)

### AD polycystic kidney disease

At least 90% of all individuals with ADPKD have a pathogenic variant in *PKD1* or *PKD2* which code for a transmembrane protein complex that regulates the transport of cations in the tubule and are not associated with ocular features. These genes are expressed at low levels in the human retina, and mouse models have no ocular phenotype. Pathogenic variants in *GANAB* and *DNAJB11* are rare causes of polycystic kidney disease and while expressed in the retina do not result in an ocular phenotype in mouse models.

### AR polycystic kidney disease

AR polycystic kidney disease (ARPKD) is caused by pathogenic variants in *PKHD1* which encodes fibrocystin, a cell membrane ciliary receptor. ARPKD is not associated with an ocular phenotype, but genome-wide association studies have recently suggested *PKHD1* as a marker for myopia [77], raised intraocular pressure, and primary open-angle glaucoma [78]. The mouse models have no ocular phenotype. A rare cause of ARPKD, *DZIP1L,* is also not expressed in the retina, and again the mouse models have no ocular phenotype.

### AD tubulointerstitial kidney disease

Pathogenic variants in *UMOD, MUC1*, and *REN* have no reported ocular associations. There is an isolated report of a unilateral coloboma and visual loss associated with a pathogenic variant in *HNF1B* (renal cysts and diabetes syndrome), a regulator of gene expression during nephron development [79], but this has not been reproduced and may be coincidental. None of these four proteins is expressed to any extent in the human retina, and none of the mouse models has ocular features.

## Ocular phenotypes in syndromic forms of cystic kidney disease

In contrast, all 6 genes associated with syndromic cystic kidney disease have ocular manifestations. These include the renal coloboma syndrome (“morning glory anomaly”); von Hippel-Lindau syndrome (retinal hemangioblastoma); tuberous sclerosis (iris and retinal hamartoma); HANAC (tortuous retinal vessels); and X-linked Alport syndrome (anterior lenticonus, corneal dystrophy, fleck retinopathy). The ocular features are distinctive and generally diagnostic.

### Renal coloboma syndrome

Inheritance is AD, and about 80% of individuals with a pathogenic variant in *PAX2,* a transcriptional regulator, have renal coloboma syndrome [80]. Variants in this gene result typically in focal and segmental glomerulosclerosis (FSGS), but congenital anomalies of the kidney and urinary tract (CAKUT) and kidney cysts also occur. Ocular associations include optic nerve dysplasia, including both optic nerve coloboma and the “morning glory” anomaly [71]. Subtle forms may be overlooked. Scleral staphyloma, chorioretinal coloboma, optic nerve cyst, microcornea, microphthalmia, macular dysplasia [81–83], nystagmus and retinal detachment [84] also occur. Iris coloboma are not a feature. The ocular abnormality is present at birth, may be bilateral, but varies in each eye and does not necessarily affect the visual fields. However, complications such as retinal detachment may occur and patients require monitoring.

### Von Hippel-Lindau disease

Von Hippel Lindau disease is an AD “tumor syndrome” that results from pathogenic variants in the *VHL* tumor suppressor gene [85]. Retinal capillary hemangiomas are present in 50 to 85% of affected individuals by the age of 25 years [85, 86, 87]. Most retinal capillary hemangiomas are due to Von Hippel-Lindau disease, and their presence prompts genetic testing [88]. Small hemangiomas do not affect vision, but larger lesions result in complications including subretinal exudation, fibrovascular proliferation, neovascular glaucoma, and retinal detachment. Regular monitoring is important to avoid loss of vision, with treatments including laser photocoagulation, photodynamic therapy, anti-VEGF injections, and vitreoretinal surgery [89–92].

### Tuberous sclerosis complex 1 and 2

Tuberous sclerosis is another AD “tumor syndrome,” caused by pathogenic variants in the *TSC1* or *TSC2* tumor suppressor genes, tuberin and hamartin, that inhibit the mammalian target of rapamycin (mTOR) pathway. Affected individuals have the characteristic retinal lesions of astrocytic hamartomas which may be flat, nodular, or transitional. Flat lesions occur in 50–70% of patients [93] and are yellow-gray retinal patches that obscure vessels. Nodular or “mulberry” lesions are calcified, elevated defects with well-defined margins found in half of all patients [94]. Transitional lesions are less common and have features of both flat and nodular hamartomas. Hamartomas rarely affect vision, but ongoing monitoring is important because of the risk of subretinal exudates and retinal detachment [95]. Hamartomas affecting the optic nerve may be mistaken for papilledema. Tuberous sclerosis also results in subependymal giant cell astrocytomas in 20% of patients that can cause papilledema [96]. Less common retinal findings include areas of peripheral hypopigmentation and chorioretinal coloboma [93]. Non-retinal findings in the eye are rare but include iris hypopigmentation [97] and juvenile cataract [98].

### Alport syndrome

Alport syndrome results from pathogenic variants in the *COL4A5, COL4A3*, or *COL4A4* genes. These contribute to the collagen IV α3α4α5 network in the basement membranes of the lens capsule, cornea, and the inner nuclear layer, and Bruch’s membrane of the retina. Kidney cysts have been reported in the XL and AD forms of Alport syndrome [16, 17]. Cysts typically occur before the age of 50 years but are few in number, and do not distort the kidney size or contribute to kidney failure [99]. With the XL and AR forms of Alport syndrome, lenticonus, central fleck retinopathy, and temporal retinal thinning are common [100], and corneal erosions and macular atrophy may occur [23]. More severe genetic variants (large rearrangements, truncating changes) are associated with more damaging ocular phenotypes [101–103]. AD Alport syndrome has no reported ocular features [104].

Kidney cysts are also found in HANAC. This results from pathogenic variants in *COL4A1* which codes for the collagen IV *a*1 chain found in blood vessel basement membranes. The cysts are usually few in number and do not result in kidney failure [105]. Retinal vessels are tortuous on imaging [74] and cerebral small vessel disease may be present [106].

## Discussion

The pediatric-onset renal ciliopathies are often associated with ocular abnormalities. These include inherited retinal degeneration, coloboma, and oculomotor disorders, but there is often variable penetrance and expression even in family members with the same disease-causing variant. The greater prevalence of ocular features in renal ciliopathies correlates with the frequent expression of the genes in the retina and the presence of ocular phenotypes in the corresponding mouse models.

In contrast, ophthalmic abnormalities are much less common or do not occur in genetic "cystic" kidney diseases that present in adulthood, namely, ADPKD, and ADTKD. ARPKD also has no ocular features. Ocular abnormalities are more common in the rarer cystic kidney syndromes such as tuberous sclerosis and von Hippel-Lindau syndrome where they affect at least half of all affected individuals. Ocular features are also found in some other cystic kidney diseases such as papillorenal syndrome and Alport syndrome. In general, the genes affected in cystic kidney disease are not expressed in the retina and most mouse models do not have an ocular phenotype.

It was generally difficult to determine how often ocular abnormalities were associated with disease due to individual genes. This was because often few individuals were reported for any gene, only some had undergone a formal ophthalmic examination and many had only been examined at presentation. However, one large well-studied cohort with Joubert syndrome had ocular motor apraxia in 80% of affected individuals, strabismus in 74%, nystagmus in 72%, ptosis in 43%, chorioretinal coloboma in 30%, optic nerve atrophy in 22%, and inherited retinal degeneration in 38% [64].

In summary, ocular features of the inherited renal ciliopathies and cystic kidney diseases may be useful diagnostically. Most forms of nephronophthisis and the other renal ciliopathies with a mainly pediatric onset have ocular features reported. Inherited retinal degeneration is a useful pointer to the diagnosis of nephronophthisis and Bardet-Biedl syndrome*.* Oculomotor disorders may be a feature of ciliopathies, especially Joubert syndrome. Coloboma suggest a diagnosis of nephronophthisis but also occur in other forms of genetic kidney disease. In contrast, ADPKD, ARPKD, and ADTKD are not associated with ocular features, but retinal hemangioma and astrocytic hamartoma suggest von Hippel-Lindau syndrome and tuberous sclerosis, respectively.

In children a complete ophthalmological examination is helpful especially where a kidney biopsy has non-specific or inconclusive findings and genetic testing is problematic. While genetic testing is increasingly available, ophthalmic examination is a fast, inexpensive, and non-invasive method that also demonstrates potentially vision-threatening disease. Furthermore, sight-threatening conditions associated with kidney disease may require ophthalmological referral for monitoring and treatment, for example, for refractive error, glaucoma, or strabismus. Monitoring and treatment is necessary for retinal hemangiomas in von Hippel-Lindau disease, astrocytic hamartoma in tuberous sclerosis, and complications of coloboma such as retinal detachment [58, 95, 107, 108]. Inherited retinal degeneration is not currently treatable, but monitoring for progression and complications is necessary [45, 109]. In the future, gene-specific therapies may be helpful. Genetic testing should be performed in individuals where these diseases are suspected.

The strengths of this study were use of the Genomics England panel for the list of genes confirmed by experts to be affected in the renal ciliopathies, the inclusion of OMIM in the clinical review, and the screening of a retinal expression database and of mouse models for ocular features. The study’s major limitations were the few reports of some diseases, and the paucity of detailed ophthalmic examinations described; the variable penetrance and expression of the genetic variants; and the lack of examination of gene expression in parts of the eye other than the retina. In addition, some of the ocular associations may have been coincidental and sometimes the ocular phenotypes may have been mild or overlooked. Mouse models do not precisely replicate human disease but the finding of a mouse ocular phenotype suggests that these features should be sought more closely in the corresponding human disease.

In conclusion, the finding of ocular abnormalities in a suspected renal ciliopathy or cystic kidney disease may confirm the disease’s genetic basis, implicate the affected gene, indicate the necessity of monitoring for ocular complications, and suggest future treatments. These ocular associations may also help us better understand the pathogenesis of the corresponding kidney disease.

### Supplementary Information

Below is the link to the electronic supplementary material.

ESM 1 (PDF 362 KB)

## Data Availability

All the data examined here is included in the manuscript, the Supplementary material or the cited databases.
