# Supplementary material for: Ocular manifestations of renal ciliopathies
Source: Pediatr Nephrol. 2023 Aug 30;39(5):1327–46. doi: 10.1007/s00467-023-06096-5 (PMC10942941; doi:10.1007/s00467-023-06096-5)
Supplement: Supplementary file 1 — ESM 1 (PDF 362 KB) [file 467_2023_6096_MOESM1_ESM.pdf]

Suppl Table 1: Ocular features in renal ciliopathies with references (based on Table 2)

These genes are from the list in the Genomics England Renal ciliopathy panel

Gene (OMIM)	Disease(s)	Ocular phenotype	Retinal expression	Mouse Phenotype
Nephronophthisis and related Renal ciliopathies				
AHI1 (608894)	Joubert syndrome 3 (608629)	Coloboma, inherited retinal degeneration oculomotor apraxia, nystagmus, strabismus [1-4], ptosis, epicanthal folds (OMIM)	**27.2 TPM**	Retinal Degeneration [5]
ALMS1 (606844)	Alstrom syndrome (203800)	Cone-rod dystrophy; pigmentary retinopathy; nystagmus; cataracts; optic neuropathy; hyperopia; constricted visual field (OMIM) [6]	**10.4 TPM**	Retinal degeneration
ANK6 (615370)	Nephronophthisis 16 (615382)	No ocular abnormalities reported in OMIM nor in search	**2.2 TPM**	None noted
ARL13B (608922)	Joubert syndrome 8 (612291)	Pigmentary retinopathy, optic disc pallor, abnormal eye movements (OMIM)	**18.2 TPM**	Retinal degeneration
ARL6 (608845)	Bardet Biedl syndrome 3 (600151)	Inherited retinal degeneration (OMIM) [7]	**46.3 TPM**	Retinal degeneration
ARMC9 (617612)	Joubert syndrome 30 (617622)	Inherited retinal degeneration, abnormal eye movements, ptosis (OMIM)	**71.5 TPM**	Retinal degeneration
B9D2 (611951)	Meckel syndrome.10, Joubert syndrome 34 (614175)	Ptosis, epicanthus, small palpebral fissures (OMIM); abnormal eye movements [8]	**1.2 TPM**	Abnormal optic cup, eye muscle morphology; aphakia
BBS1 (209901)	Bardet Biedl syndrome 1 (209900)	Rod-cone dystrophy, strabismus, cataracts (OMIM); Inherited retinal degeneration. For all BBS [9-27]	**21.4 TPM**	Retinal degeneration, anophthalmia
BBS10 (610148)	Bardet Biedl syndrome 10 (615987)	Inherited retinal degeneration [9-27]	**11.4 TPM**	Retinal degeneration
BBS12 (610683)	Bardet Biedl syndrome 12 (615989)	Inherited retinal degeneration [9-27]	**17.6 TPM**	Retinal degeneration
BBS2 (606151)	Bardet Biedl syndrome 2 (615981)	Inherited retinal degeneration [9-27]	**86.5 TPM**	Retinal degeneration
BBS4 (600374)	Bardet Biedl syndrome 4 (615982)	Inherited retinal degeneration [9-27]	**45.4 TPM**	Retinal degeneration, optic nerve atrophy
BBS5 (603650)	Bardet Biedl syndrome 5 (615983)	Inherited retinal degeneration [9-27]	**11.3 TPM**	Retinal degeneration
BBS7 (607590)	Bardet Biedl syndrome 7 (615984)	Inherited retinal degeneration [9-27]	**56.6 TPM**	Retinal degeneration, abnormal lens
BBS9 (607968)	Bardet Biedl Syndrome 9 (615986)	Inherited retinal degeneration [28], cataract, optic nerve dysplasia, nystagmus	**21.9 TPM**	None noted
BBIP1 (613605)	Bardet Biedl syndrome 18 (615995)	Inherited retinal degeneration [9-27], cataracts	**50.1 TPM**	Retinal degeneration
C5orf42 (614571)	Joubert syndrome 17 (614615); Orofaciodigital syndrome VI (277170)	Oculomotor apraxia (OMIM); Hypertelorism, epicanthal folds, nystagmus, oculomotor apraxia [29]	**5.7 TPM**	Abnormal eye morphology
CC2D2A (612013)	Joubert 9 (612285), Meckel syndrome 6 (612284), COACH syndrome 2 (619111)	Astigmatism, coloboma, inherited retinal degeneration, oculomotor apraxia, nystagmus [30-32], cataract (OMIM)	**84.7 TPM**	Anophthalmia[33], microphthalmia, retinal degeneration
CENPF (600236)	Stromme syndrome (243605)	Microophthalmia, microcornea, anterior chamber defects, iris coloboma, optic nerve hypoplasia, cataracts, hypertelorism, tortuous retinal vessels [34]	**1.0 TPM**	None noted
CEP104 (616690)	Joubert syndrome 25 (616781)	Oculomotor apraxia (OMIM), nystagmus, inherited retinal degeneration	**9.0 TPM**	None noted
CEP164 (614848)	Nephronophthisis 15 (614845)	Inherited retinal degeneration[35]; Leber congenital amaurosis, nystagmus (OMIM)	**26 TPM**	None noted

CEP290 ***(***610142)	Nephronophthisis 6 , Joubert 5 (610188), Bardet Biedl 14 (615991), Meckel syndrome 4 (611134)	Inherited retinal degeneration, Coats-like exudative vasculopathy [36, 37]; congenital amaurosis, nystagmus, retinal coloboma; oculomotor apraxia (OMIM)	**7.2 TPM**	Retinal degeneration[38]
CEP41 ***(***610523)	Joubert syndrome 15 (614464)	Chorioretinal coloboma, inherited retinal degeneration, oculomotor apraxia (OMIM) [39]	**7.7 TPM**	None noted
CEP83 ***(***615847)	Nephronophthisis 18 (615862)	Inherited retinal degeneration, strabismus [40]	**5.3 TPM**	None noted
CRB2 **(609720)**	Ventriculomegaly with cystic kidney disease (219730)	Inherited retinal degeneration [41]	**8.1 TPM; high in limiting membrane**	Inherited retinal degeneration
CSPP1 **(611654)**	Joubert syndrome 21 (61536)	Inherited retinal degeneration Oculomotor apraxia, strabismus, ptosis, fused eyes, anophthalmia (OMIM); nystagmus, corneal clouding (rare), cataracts (rare) [42, 43]	**2.1 TPM**	None noted
DDX59 (615464)	Oral-Facial-digital syndrome V (174300)	Epicanthus, hypertelorism, telecanthus (OMIM), coloboma, ptosis [44]	**17.4 TPM**	None noted
DHCR7 **(602858)**	Smith-Lemli-Opitz syndrome (270400)	Ptosis, epicanthal folds, cataracts, hypertelorism, strabismus (OMIM); optic atrophy, blepharoptosis, optic nerve hypoplasia [45]	**8.7 TPM**	Microphthalmia
DYNC2H1 ***(***603297)	Jeune syndrome 3	Inherited retinal degeneration [46]	**10.5 TPM**	Abnormal eye morphology
HYLS1 **(610693)**	Hydrolethalus syndrome (236680)	Micropthalmia (OMIM); optic nerve coloboma and hypoplasia [45]	**4.3 TPM**	None noted
ICK **(612325)**	Endocrine-cerebro-osteodysplasia (612651)	Small sunken eyes, fused eye lids (OMIM)	**NA**	None noted
IFT122 ***(606045)***	Cranioectodermal dysplasia 1 (218330)	Hypertelorism, epicanthal folds, myopia, nystagmus, inherited retinal degeneration [47] (OMIM)	**11.2 TPM**	Abnormal eye morphology
IFT43 ***(***614068)	Cranioectodermal dysplasia 3 (614099), Jeune syndrome 18 (617866)	Inherited retinal degeneration [48]	**25.1 TPM**	None noted
INPP5E **(613037)**	Joubert syndrome 1 (613037)	Inherited retinal degeneration [49]; abnormal jerky eye movements, oculomotor apraxia [50], coloboma of the optic nerve; chorioretinal coloboma [51], epicanthal folds, ptosis (OMIM)	**7.4 TPM**	Retinal degener-ation; microphthalmia, abnormal eye morphology
INVS ***(***243305)	Nephronophthisis 2 (602088)	Inherited retinal degeneration [52]	**11.8 TPM**	None noted
IQCB1 ***(609237)***	Senior-Loken syndrome 5 (609254)	Leber congenital amaurosis; inherited retinal degeneration (OMIM)	**18.9 TPM; medium in photo-receptor cells**	Increased corneal thickness
KIAA0586 **(610178*)***	Joubert syndrome 23 (616490)	Inherited retinal degeneration, nystagmus [53]; abnormal eye movements, coloboma (OMIM)	**7.9 TPM**	None noted
KIAA0753 **(617112)**	Joubert syndrome 38 (619476)	Inherited retinal degeneration, nystagmus, abnormal eye movements [54], epicanthal folds, oculomotor apraxia (OMIM)	**10.8 TPM**	None noted
KIF7 **(611254)**	Joubert syndrome 7 (200990)	Strabismus, hypertelorism, epicanthal folds, optic atrophy, inherited retinal degeneration, nystagmus, coloboma (OMIM) [55]	**0.2 TPM**	Microphthalmia, anophthalmia
LZTFL1 ***(***606568)	Bardet Biedl syndrome 17 (615994)	Inherited retinal degeneration (OMIM)	**15.8 TPM**	Retinal degeneration
MAPKBP1 ***(616786)***	Nephronophthisis 20 (617271)	No ocular features reported nor in OMIM	**9.0 TPM**	None noted
MKKS ***(604896)***	Bardet-Biedl syndrome (605231); McKusick-	Inherited retinal degeneration [56]	**22.8 TPM**	Retinal degeneration

	Kaufman syndrome (236700)			
MKS1 *(*609883)	Meckel 1 (249000); Bardet Biedl 13 (615990); Joubert syndrome 28 (617121)	Oculomotor apraxia, nystagmus, microphthalmia, Inherited retinal degeneration[57-59], coloboma, optic disc pallor, ptosis	**6.1 TPM**	Anophthalmia[60]; microphthalmia, abnormal eye morphology
NEK8 ***(609799)***	Nephronophthisis 9 (613824); Renal-hepatic pancreatic dysplasia 2 (615415)	No ocular features reported nor in OMIM	**0.6 TPM**	None noted
NPHP1 *(*607100)	Nephronophthisis 1 (256100), Joubert Syndrome 4 (609583), Senior-Loken syndrome1 (266900)	Inherited retinal degeneration [61, 62], Stargardt-like retinopathy[63], oculomotor apraxia [64]	**10.8 TPM**	Retinal degeneration [65]
NPHP3 (608002)	Nephronophthisis 3 (604387), Meckel syndrome 7 (267010)	Inherited retinal degeneration[66], cataract, nystagmus	**3.1 TPM**	None noted
NPHP4 ***(6***07215)	Nephronophthisis 4 (606966), Senior-Loken syndrome (606966)	Coloboma, inherited retinal degeneration, oculomotor apraxia [35, 67]; amblyopia, rotary nystagmus (OMIM)	**2.4 TPM**	Retinal degeneration [68]
OFD-1 *(*300170)	Orofaciodigital Syndrome 1 (311200)	Inherited retinal degeneration, bilateral idiopathic demyelinating optic neuritis [69]; epicanthal folds (OMIM)	**11.2 TPM**	None noted
PMM2 ***(601785)***	Congenital disorder of glycosylation 1a (212065)	Abnormal eye movements [70], strabismus, nystagmus, inherited retinal degeneration (OMIM)	**1.5 TPM**	Abnormal eye morphology
RPGRIP1L ***(6***605446)	Nephronophthisis 8, Joubert 7 (611560), Meckel 5 (611561)	Coloboma, inherited retinal degeneration, oculomotor apraxia [71-73]; nystagmus, ptosis (OMIM)	**2.9 TPM**	Anophthalmia [74]; abnormal optic cup; eye muscles
SDCCAG8 *(*613524)	NPHP10, Bardet Biedl 16	Inherited retinal degeneration [75, 76]	**3.7 TPM**	Retinal degeneration [77]
TCTN1 ***(609863)***	Joubert syndrome 13 (614173)	No ocular features reported nor in OMIM	**3.0 TPM**	None reported
TCTN2 *(*613846)	Joubert syndrome 24 (616654)	Anophthalmia, nystagmus [78, 79]	**17.6 TPM**	Anophthalmia[80]; microphthalmia
TCTN3 (613847)	Joubert 18 (614815); Orofaciodigital syndrome IV (258860)	Abnormal eye movements [81], hypertelorism, epicanthal folds (OMIM)	**22.2TPM**	Anophthalmia)
TMEM107 ***(616183)***	Meckel syndrome 13 (617562); Joubert syndrome (617562), Orofaciodigital syndrome XVI (617563)	Inherited retinal degeneration, oculomotor apraxia, ptosis (OMIM)	**29.6 TPM**	Microphthalmia (MGI)
TMEM138 *(*614459)	Joubert syndrome 16 (614465)	Coloboma, inherited retinal degeneration, oculomotor apraxia, nystagmus, strabismus[82]	**50.7 TPM**	None noted
TMEM216 *(*613277)	Joubert 2 (608091), Meckel syndrome 2 (603194)	Coloboma, inherited retinal degeneration, oculomotor apraxia, nystagmus, strabismus (esotropia)[83]	**11.8 TPM**	None noted
TMEM231 *(*614949)	Joubert 20 (614949), Meckel 11 (615397)	Inherited retinal degeneration, oculomotor apraxia [84, 85]	**5.2 TPM**	Anophthalmia[86]; microphthalmia
TMEM237 *(*614423)	Joubert syndrome 14 (614424)	Morning glory anomaly, retinal coloboma, nystagmus, strabismus [87]; hypertelorism, ptosis, epicanthal folds (OMIM)	**89.3 TPM**	None noted
TMEM67 *(*609884)	Nephronophthisis 11 (613550), Joubert 6 (610688), Meckel 3 (607361)	Ptosis, anisocoria, chorioretinal coloboma, inherited retinal degeneration, oculomotor apraxia, nystagmus [35, 88, 89]	**6.1TPM**	Retinal degeneration [90]
TRAF3IP1 ***(607380)***	Senior Loken syndrome 9 (616629)	Inherited retinal degeneration, nystagmus, strabismus (OMIM); iris patterns [91]	**6.7 TPM**	Microphthalmia, thick cornea

TTC21B ***(***612014)	NPHP12, Jeune syndrome 4	Pathological myopia associated with chorioretinal atrophy, choroidal neovascularisation and traction retinopathy [92]	**7.5 TPM**	Shortened primary cilia[93]
TTC8 ***(***608132)	Bardet Biedl syndrome 8 (613464)	High myopia, inherited retinal degeneration, optic neuropathy (OMIM)	**37.4 TPM**	Retinal degeneration
TXNDC15 (619879)	Meckel syndrome 14 (619879)	Hypertelorism, microphthalmia (OMIM)	**38.5 TPM**	None noted
WDPCP (613580)	Bardet Biedl syndrome	Inherited retinal degeneration [94]	**12.2 TPM**	Anophthalmia[95]
WDR19 (608151)	NPHP13, Jeune 5, Cranioectodermal dysplasia 4	Inherited retinal degeneration, nystagmus [96-98]	**13.0 TPM**	Anophthalmia [99]
WDR35 (613602)	Cranioectodermal dysplasia 2, Jeune 7	Optic nerve coloboma, nystagmus, hypermetropia, strabismus, amblyopia [100-102]	**12.6 TPM**	None noted
WDR60 (615462)	Jeune syndrome	Inherited retinal degeneration (OMIM)	**14.1 TPM**	None noted

References

1. Parisi MA, Doherty D, Eckert ML, Shaw DW, Ozyurek H, Aysun S, Giray O, Al Swaid A, Al Shahwan S, Dohayan N, Bakhsh E, Indridason OS, Dobyns WB, Bennett CL, Chance PF, Glass IA (2006) *AHI1* mutations cause both retinal dystrophy and renal cystic disease in Joubert syndrome. J Med Genet 43:334-339
2. Lagier-Tourenne C, Boltshauser E, Breivik N, Gribaa M, Bétard C, Barbot C, Koenig M (2004) Homozygosity mapping of a third Joubert syndrome locus to 6q23. J Med Genet 41:273-277
3. Salva I, Albuquerque C, Moreira A, Dâmaso C (2016) Nystagmus in a newborn: a manifestation of Joubert syndrome in the neonatal period. BMJ Case Rep 2016
4. Nguyen TT, Hull S, Roepman R, van den Born LI, Oud MM, de Vrieze E, Hetterschijt L, Letteboer SJF, van Beersum SEC, Blokland EA, Yntema HG, Cremers FPM, van der Zwaag PA, Arno G, van Wijk E, Webster AR, Haer-Wigman L (2017) Missense mutations in the WD40 domain of *AHI1* cause non-syndromic retinitis pigmentosa. J Med Genet 54:624-632
5. Louie CM, Caridi G, Lopes VS, Brancati F, Kispert A, Lancaster MA, Schlossman AM, Otto EA, Leitges M, Gröne H-J, Lopez I, Gudiseva HV, O'Toole JF, Vallespin E, Ayyagari R, Ayuso C, Cremers FPM, den Hollander AI, Koenekoop RK, Dallapiccola B, Ghiggeri GM, Hildebrandt F, Valente EM, Williams DS, Gleeson JG (2010) *AHI1 i*s required for photoreceptor outer segment development and is a modifier for retinal degeneration in nephronophthisis. Nat Genet 42:175-180
6. Tang VD, Egense A, Yiu G, Meyers E, Moshiri A, Shankar SP (2022) Retinal dystrophies: A look beyond the eyes. Am J Ophthalmol Case Rep 27:101613
7. Biswas P, Duncan JL, Maranhao B, Kozak I, Branham K, Gabriel L, Lin JH, Barteselli G, Navani M, Suk J, Parke M, Schlechter C, Weleber RG, Heckenlively JR, Dagnelie G, Lee P, Riazuddin SA, Ayyagari R (2017) Genetic analysis of 10 pedigrees with inherited retinal degeneration by exome sequencing and phenotype-genotype association. Physiol Genomics 49:216-229
8. Parisi M, Glass I (1993) Joubert Syndrome. In: Adam MP, Everman DB, Mirzaa GM, Pagon RA, Wallace SE, Bean LJH, Gripp KW, Amemiya A (eds) GeneReviews ((R)), Seattle (WA)
9. Khan SA, Muhammad N, Khan MA, Kamal A, Rehman ZU, Khan S (2016) Genetics of human Bardet-Biedl syndrome, an updates. Clin Genet 90:3-15
10. Khan S, Ullah I, Irfanullah, Touseef M, Basit S, Khan MN, Ahmad W (2013) Novel homozygous mutations in the genes *ARL6* and *BBS10* underlying Bardet-Biedl syndrome. Gene 515:84-88
11. Gouronc A, Zilliox V, Jacquemont ML, Darcel F, Leuvrey AS, Nourisson E, Antin M, Alessandri JL, Doray B, Gueguen P, Payet F, Randrianaivo H, Stoetzel C, Scheidecker S, Flodrops H, Dollfus H, Muller J (2020) High prevalence of Bardet-Biedl syndrome in La Réunion Island is due to a founder variant in *ARL6/BBS3*. Clin Genet 98:166-171
12. Zenteno JC, García-Montaño LA, Cruz-Aguilar M, Ronquillo J, Rodas-Serrano A, Aguilar-Castul L, Matsui R, Vencedor-Meraz CI, Arce-González R, Graue-Wiechers F, Gutiérrez-Paz M, Urrea-Victoria T, de Dios Cuadras U, Chacón-Camacho OF (2020) Extensive genic and allelic heterogeneity underlying inherited retinal dystrophies in Mexican patients molecularly analyzed by next-generation sequencing. Mol Genet Genomic Med 8.
13. Iannaccone A, Mykytyn K, Persico AM, Searby CC, Baldi A, Jablonski MM, Sheffield VC (2005) Clinical evidence of decreased olfaction in Bardet-Biedl syndrome caused by a deletion in the *BBS4* gene. Am J Med Genet A 132a:343-346
14. Schaefer E, Lauer J, Durand M, Pelletier V, Obringer C, Claussmann A, Braun JJ, Redin C, Mathis C, Muller J, Schmidt-Mutter C, Flori E, Marion V, Stoetzel C, Dollfus H (2014) Mesoaxial polydactyly is a major feature in Bardet-Biedl syndrome patients with *LZTFL1* (BBS17) mutations. Clin Genet 85:476-481
15. Scheidecker S, Hull S, Perdomo Y, Studer F, Pelletier V, Muller J, Stoetzel C, Schaefer E, Defoort-Dhellemmes S, Drumare I, Holder GE, Hamel CP, Webster AR, Moore AT, Puech B, Dollfus HJ (2015) Predominantly Cone-System Dysfunction as Rare Form of Retinal Degeneration in Patients With

Molecularly Confirmed Bardet-Biedl Syndrome. Am J Ophthalmol 160:364-372.e361
16. Young TL, Penney L, Woods MO, Parfrey PS, Green JS, Hefferton D, Davidson WS (1999) A fifth locus for Bardet-Biedl syndrome maps to chromosome 2q31. Am J Hum Genet 64:900-904
17. Harville HM, Held S, Diaz-Font A, Davis EE, Diplas BH, Lewis RA, Borochowitz ZU, Zhou W, Chaki M, MacDonald J, Kayserili H, Beales PL, Katsanis N, Otto E, Hildebrandt F (2010) Identification of 11 novel mutations in eight BBS genes by high-resolution homozygosity mapping. J Med Genet 47:262-267
18. Scheidecker S, Etard C, Pierce NW, Geoffroy V, Schaefer E, Muller J, Chennen K, Flori E, Pelletier V, Poch O, Marion V, Stoetzel C, Strähle U, Nachury MV, Dollfus H (2014) Exome sequencing of Bardet-Biedl syndrome patient identifies a null mutation in the BBSome subunit BBIP1 (BBS18). J Med Genet 51:132-136
19. Kulaga HM, Leitch CC, Eichers ER, Badano JL, Lesemann A, Hoskins BE, Lupski JR, Beales PL, Reed RR, Katsanis N (2004) Loss of BBS proteins causes anosmia in humans and defects in olfactory cilia structure and function in the mouse. Nat Genet 36:994-998
20. Nishimura DY, Fath M, Mullins RF, Searby C, Andrews M, Davis R, Andorf JL, Mykytyn K, Swiderski RE, Yang B, Carmi R, Stone EM, Sheffield VC (2004) *Bbs2*-null mice have neurosensory deficits, a defect in social dominance, and retinopathy associated with mislocalization of rhodopsin. Proc Natl Acad Sci U S A 101:16588-16593
21. Zhang Q, Nishimura D, Seo S, Vogel T, Morgan DA, Searby C, Bugge K, Stone EM, Rahmouni K, Sheffield VC (2011) Bardet-Biedl syndrome 3 (Bbs3) knockout mouse model reveals common BBS-associated phenotypes and Bbs3 unique phenotypes. Proc Natl Acad Sci U S A 108:20678-20683
22. Tadenev AL, Kulaga HM, May-Simera HL, Kelley MW, Katsanis N, Reed RR (2011) Loss of Bardet-Biedl syndrome protein-8 (BBS8) perturbs olfactory function, protein localization, and axon targeting. Proc Natl Acad Sci U S A 108:10320-10325
23. Datta P, Allamargot C, Hudson JS, Andersen EK, Bhattarai S, Drack AV, Sheffield VC, Seo S (2015) Accumulation of non-outer segment proteins in the outer segment underlies photoreceptor degeneration in Bardet-Biedl syndrome. Proc Natl Acad Sci U S A 112:E4400-4409
24. Meehan TF, Conte N, West DB, Jacobsen JO, Mason J, Warren J, Chen CK, Tudose I, Relac M, Matthews P, Karp N, Santos L, Fiegel T, Ring N, Westerberg H, Greenaway S, Sneddon D, Morgan H, Codner GF, Stewart ME, Brown J, Horner N, Haendel M, Washington N, Mungall CJ, Reynolds CL, Gallegos J, Gailus-Durner V, Sorg T, Pavlovic G, Bower LR, Moore M, Morse I, Gao X, Tocchini-Valentini GP, Obata Y, Cho SY, Seong JK, Seavitt J, Beaudet AL, Dickinson ME, Herault Y, Wurst W, de Angelis MH, Lloyd KCK, Flenniken AM, Nutter LMJ, Newbigging S, McKerlie C, Justice MJ, Murray SA, Svenson KL, Braun RE, White JK, Bradley A, Flicek P, Wells S, Skarnes WC, Adams DJ, Parkinson H, Mallon AM, Brown SDM, Smedley D (2017) Disease model discovery from 3,328 gene knockouts by The International Mouse Phenotyping Consortium. Nat Genet 49:1231-1238
25. Zhang Q, Nishimura D, Vogel T, Shao J, Swiderski R, Yin T, Searby C, Carter CS, Kim G, Bugge K, Stone EM, Sheffield VC (2013) BBS7 is required for BBSome formation and its absence in mice results in Bardet-Biedl syndrome phenotypes and selective abnormalities in membrane protein trafficking. J Cell Sci 126:2372-2380.
26. Loktev AV, Jackson PK (2013) Neuropeptide Y family receptors traffic via the Bardet-Biedl syndrome pathway to signal in neuronal primary cilia. Cell Rep 5:1316-1329
27. Cognard N, Scerbo MJ, Obringer C, Yu X, Costa F, Haser E, Le D, Stoetzel C, Roux MJ, Moulin B, Dollfus H, Marion V (2015) Comparing the Bbs10 complete knockout phenotype with a specific renal epithelial knockout one highlights the link between renal defects and systemic inactivation in mice. Cilia 4:10
28. Abu-Safieh L, Al-Anazi S, Al-Abdi L, Hashem M, Alkuraya H, Alamr M, Sirelkhatim MO, Al-Hassnan Z, Alkuraya B, Mohamed JY, Al-Salem A, Alrashed M, Faqeih E, Softah A, Al-Hashem A, Wali S, Rahbeeni Z, Alsayed M, Khan AO, Al-Gazali L, Taschner PE, Al-Hazzaa S, Alkuraya FS (2012) In search of triallelism in Bardet-Biedl syndrome. Eur J Hum Genet 20:420-427
29. Srour M, Schwartzentruber J, Hamdan FF, Ospina LH, Patry L, Labuda D, Massicotte C, Dobrzeniecka S, Capo-Chichi JM, Papillon-Cavanagh S, Samuels ME, Boycott KM, Shevell MI, Laframboise R, Desilets V, Consortium FC, Maranda B, Rouleau GA, Majewski J, Michaud JL (2012) Mutations in *C5ORF42* cause

Joubert syndrome in the French Canadian population. Am J Hum Genet 90:693-700
30. Noor A, Windpassinger C, Patel M, Stachowiak B, Mikhailov A, Azam M, Irfan M, Siddiqui ZK, Naeem F, Paterson AD, Lutfullah M, Vincent JB, Ayub M (2008) *CC2D2A*, encoding a coiled-coil and C2 domain protein, causes autosomal-recessive mental retardation with retinitis pigmentosa. Am J Hum Genet 82:1011-1018
31. Gorden NT, Arts HH, Parisi MA, Coene KL, Letteboer SJ, van Beersum SE, Mans DA, Hikida A, Eckert M, Knutzen D, Alswaid AF, Ozyurek H, Dibooglu S, Otto EA, Liu Y, Davis EE, Hutter CM, Bammler TK, Farin FM, Dorschner M, Topçu M, Zackai EH, Rosenthal P, Owens KN, Katsanis N, Vincent JB, Hildebrandt F, Rubel EW, Raible DW, Knoers NV, Chance PF, Roepman R, Moens CB, Glass IA, Doherty D (2008) *CC2D2A* is mutated in Joubert syndrome and interacts with the ciliopathy-associated basal body protein CEP290. Am J Hum Genet 83:559-571
32. Doherty D, Parisi MA, Finn LS, Gunay-Aygun M, Al-Mateen M, Bates D, Clericuzio C, Demir H, Dorschner M, van Essen AJ, Gahl WA, Gentile M, Gorden NT, Hikida A, Knutzen D, Ozyurek H, Phelps I, Rosenthal P, Verloes A, Weigand H, Chance PF, Dobyns WB, Glass IA (2010) Mutations in 3 genes (*MKS3, CC2D2A* and *RPGRIP1L*) cause COACH syndrome (Joubert syndrome with congenital hepatic fibrosis). J Med Genet 47:8-21
33. Veleri S, Manjunath SH, Fariss RN, May-Simera H, Brooks M, Foskett TA, Gao C, Longo TA, Liu P, Nagashima K, Rachel RA, Li T, Dong L, Swaroop A (2014) Ciliopathy-associated gene *Cc2d2a* promotes assembly of subdistal appendages on the mother centriole during cilia biogenesis. Nature Communications 5:4207
34. Filges I, Stromme P (2020) CUGC for Stromme syndrome and CENPF-related disorders. Eur J Hum Genet 28:132-136
35. Chaki M, Hoefele J, Allen SJ, Ramaswami G, Janssen S, Bergmann C, Heckenlively JR, Otto EA, Hildebrandt F (2011) Genotype-phenotype correlation in 440 patients with NPHP-related ciliopathies. Kidney Int 80:1239-1245
36. Feldhaus B, Weisschuh N, Nasser F, den Hollander AI, Cremers FPM, Zrenner E, Kohl S, Zobor D (2020) *CEP290* Mutation Spectrum and Delineation of the Associated Phenotype in a Large German Cohort: A Monocentric Study. Am J Ophthalmol 211:142-150
37. Moloney TP, Patel C, Gole GA (2014) Exudative vasculopathy in a child with Leber congenital amaurosis. J AAPOS 18:297-299
38. Chang B, Khanna H, Hawes N, Jimeno D, He S, Lillo C, Parapuram SK, Cheng H, Scott A, Hurd RE (2006) In-frame deletion in a novel centrosomal/ciliary protein CEP290/NPHP6 perturbs its interaction with RPGR and results in early-onset retinal degeneration in the rd16 mouse. Hum Mol Genet 15:1847-1857
39. Lee JE, Silhavy JL, Zaki MS, Schroth J, Bielas SL, Marsh SE, Olvera J, Brancati F, Iannicelli M, Ikegami K, Schlossman AM, Merriman B, Attié-Bitach T, Logan CV, Glass IA, Cluckey A, Louie CM, Lee JH, Raynes HR, Rapin I, Castroviejo IP, Setou M, Barbot C, Boltshauser E, Nelson SF, Hildebrandt F, Johnson CA, Doherty DA, Valente EM, Gleeson JG (2012) *CEP41* is mutated in Joubert syndrome and is required for tubulin glutamylation at the cilium. Nat Genet 44:193-199
40. Failler M, Gee HY, Krug P, Joo K, Halbritter J, Belkacem L, Filhol E, Porath JD, Braun DA, Schueler M, Frigo A, Alibeu O, Masson C, Brochard K, Hurault de Ligny B, Novo R, Pietrement C, Kayserili H, Salomon R, Gubler MC, Otto EA, Antignac C, Kim J, Benmerah A, Hildebrandt F, Saunier S (2014) Mutations of *CEP83* cause infantile nephronophthisis and intellectual disability. Am J Hum Genet 94:905-914
41. Grudzinska Pechhacker MK, Di Scipio M, Vig A, Tumber A, Roslin N, Tavares E, Vincent A, Heon E (2020) *CRB1*-related retinopathy overlapping the ocular phenotype of S-adenosylhomocysteine hydrolase deficiency. Ophthalmic Genet 41:457-464
42. Tuz K, Bachmann-Gagescu R, O'Day DR, Hua K, Isabella CR, Phelps IG, Stolarski AE, O'Roak BJ, Dempsey JC, Lourenco C, Alswaid A, Bonnemann CG, Medne L, Nampoothiri S, Stark Z, Leventer RJ, Topcu M, Cansu A, Jagadeesh S, Done S, Ishak GE, Glass IA, Shendure J, Neuhauss SC, Haldeman-Englert CR, Doherty D, Ferland RJ (2014) Mutations in *CSPP1* cause primary cilia abnormalities and Joubert syndrome with or without Jeune asphyxiating thoracic dystrophy. Am J Hum Genet 94:62-72

43. Akizu N, Silhavy JL, Rosti RO, Scott E, Fenstermaker AG, Schroth J, Zaki MS, Sanchez H, Gupta N, Kabra M, Kara M, Ben-Omran T, Rosti B, Guemez-Gamboa A, Spencer E, Pan R, Cai N, Abdellateef M, Gabriel S, Halbritter J, Hildebrandt F, van Bokhoven H, Gunel M, Gleeson JG (2014) Mutations in *CSPP1* lead to classical Joubert syndrome. Am J Hum Genet 94:80-86
44. Faily S, Perveen R, Urquhart J, Chandler K, Clayton-Smith J (2017) Confirmation that mutations in *DDX59* cause an autosomal recessive form of oral-facial-digital syndrome: Further delineation of the DDX59 phenotype in two new families. Eur J Med Genet 60:527-532
45. Atchaneeyasakul LO, Linck LM, Connor WE, Weleber RG, Steiner RD (1998) Eye findings in 8 children and a spontaneously aborted fetus with RSH/Smith-Lemli-Opitz syndrome. Am J Med Genet 80:501-505
46. Vig A, Poulter JA, Ottaviani D, Tavares E, Toropova K, Tracewska AM, Mollica A, Kang J, Kehelwathugoda O, Paton T, Maynes JT, Wheway G, Arno G, Khan KN, McKibbin M, Toomes C, Ali M, Di Scipio M, Li S, Ellingford J, Black G, Webster A, Rydzanicz M, Stawiński P, Płoski R, Vincent A, Cheetham ME, Inglehearn CF, Roberts A, Heon E (2020) *DYNC2H1* hypomorphic or retina-predominant variants cause nonsyndromic retinal degeneration. Genet Med 22:2041-2051
47. Tan W, Lin A, Keppler-Noreuil K (1993) Cranioectodermal Dysplasia. In: Adam MP, Everman DB, Mirzaa GM, Pagon RA, Wallace SE, Bean LJH, Gripp KW, Amemiya A (eds) GeneReviews Seattle (WA)
48. Biswas P, Duncan JL, Ali M, Matsui H, Naeem MA, Raghavendra PB, Frazer KA, Arts HH, Riazuddin S, Akram J, Hejtmancik JF, Riazuddin SA, Ayyagari R (2017) A mutation in *IFT43* causes non-syndromic recessive retinal degeneration. Hum Mol Genet 26:4741-4751
49. Saraiva JM, Baraitser M (1992) Joubert syndrome: a review. Am J Med Genet 43:726-731
50. Maria BL, Boltshauser E, Palmer SC, Tran TX (1999) Clinical features and revised diagnostic criteria in Joubert syndrome. J Child Neurol 14:583-591
51. Lindhout D, Barth PG, Valk J, Boen-Tan TN (1980) The Joubert syndrome associated with bilateral chorioretinal coloboma. Eur J Pediatr 134:173-176
52. O'Toole JF, Otto EA, Frishberg Y, Hildebrandt F (2006) Retinitis pigmentosa and renal failure in a patient with mutations in *INVS*. Nephrol Dial Transplant 21:1989-1991
53. Bachmann-Gagescu R, Phelps IG, Dempsey JC, Sharma VA, Ishak GE, Boyle EA, Wilson M, Marques Lourenco C, Arslan M, University of Washington Center for Mendelian G, Shendure J, Doherty D (2015) *KIAA0586* is Mutated in Joubert Syndrome. Hum Mutat 36:831-835
54. Stephen J, Vilboux T, Mian L, Kuptanon C, Sinclair CM, Yildirimli D, Maynard DM, Bryant J, Fischer R, Vemulapalli M, Mullikin JC, Program NCS, Huizing M, Gahl WA, Malicdan MCV, Gunay-Aygun M (2017) Mutations in *KIAA0753* cause Joubert syndrome associated with growth hormone deficiency. Hum Genet 136:399-408
55. Putoux A, Thomas S, Coene KL, Davis EE, Alanay Y, Ogur G, Uz E, Buzas D, Gomes C, Patrier S, Bennett CL, Elkhartoufi N, Frison MH, Rigonnot L, Joye N, Pruvost S, Utine GE, Boduroglu K, Nitschke P, Fertitta L, Thauvin-Robinet C, Munnich A, Cormier-Daire V, Hennekam R, Colin E, Akarsu NA, Bole-Feysot C, Cagnard N, Schmitt A, Goudin N, Lyonnet S, Encha-Razavi F, Siffroi JP, Winey M, Katsanis N, Gonzales M, Vekemans M, Beales PL, Attie-Bitach T (2011) *KIF7* mutations cause fetal hydrolethalus and acrocallosal syndromes. Nat Genet 43:601-606
56. Hulleman JD, Nguyen A, Ramprasad VL, Murugan S, Gupta R, Mahindrakar A, Angara R, Sankurathri C, Mootha VV (2016) A novel H395R mutation in MKKS/BBS6 causes retinitis pigmentosa and polydactyly without other findings of Bardet-Biedl or McKusick-Kaufman syndrome. Mol Vis 22:73-81
57. Leitch CC, Zaghloul NA, Davis EE, Stoetzel C, Diaz-Font A, Rix S, Alfadhel M, Lewis RA, Eyaid W, Banin E, Dollfus H, Beales PL, Badano JL, Katsanis N (2008) Hypomorphic mutations in syndromic encephalocele genes are associated with Bardet-Biedl syndrome. Nat Genet 40:443-448
58. Xing DJ, Zhang HX, Huang N, Wu KC, Huang XF, Huang F, Tong Y, Pang CP, Qu J, Jin ZB (2014) Comprehensive molecular diagnosis of Bardet-Biedl syndrome by high-throughput targeted exome sequencing. PLoS One 9:e90599
59. Romani M, Micalizzi A, Valente EM (2013) Joubert syndrome: congenital cerebellar ataxia with the molar tooth. The Lancet Neurology 12:894-905

60. Cui C, Chatterjee B, Francis D, Yu Q, SanAgustin JT, Francis R, Tansey T, Henry C, Wang B, Lemley B, Pazour GJ, Lo CW (2011) Disruption of Mks1 localization to the mother centriole causes cilia defects and developmental malformations in Meckel-Gruber syndrome. Dis Model Mech 4:43-56
61. Ronquillo CC, Bernstein PS, Baehr W (2012) Senior-Løken syndrome: a syndromic form of retinal dystrophy associated with nephronophthisis. Vision Res 75:88-97
62. Birtel J, Eisenberger T, Gliem M, Müller PL, Herrmann P, Betz C, Zahnleiter D, Neuhaus C, Lenzner S, Holz FG, Mangold E, Bolz HJ, Charbel Issa P (2018) Clinical and genetic characteristics of 251 consecutive patients with macular and cone/cone-rod dystrophy. Sci Rep 8:4824
63. Kang HG, Ahn YH, Kim JH, Ha IS, Yu YS, Park YH, Cheong HI (2015) Atypical retinopathy in patients with nephronophthisis type 1: an uncommon ophthalmological finding. Clin Exp Ophthalmol 43:437-442
64. Deacon BS, Lowery RS, Phillips PH, Schaefer GB (2013) Congenital ocular motor apraxia, the NPHP1 gene, and surveillance for nephronophthisis. J AAPOS 17:332-333
65. Jiang ST, Chiou YY, Wang E, Chien YL, Ho HH, Tsai FJ, Lin CY, Tsai SP, Li H (2009) Essential role of nephrocystin in photoreceptor intraflagellar transport in mouse. Hum Mol Genet 18:1566-1577
66. Omran H, Sasmaz G, Häffner K, Volz A, Olbrich H, Melkaoui R, Otto E, Wienker TF, Korinthenberg R, Brandis M, Antignac C, Hildebrandt F (2002) Identification of a gene locus for Senior-Løken syndrome in the region of the nephronophthisis type 3 gene. J Am Soc Nephrol 13:75-79
67. Schuermann MJ, Otto E, Becker A, Saar K, Rüschendorf F, Polak BC, Ala-Mello S, Hoefele J, Wiedensohler A, Haller M, Omran H, Nürnberg P, Hildebrandt F (2002) Mapping of gene loci for nephronophthisis type 4 and Senior-Løken syndrome, to chromosome 1p36. Am J Hum Genet 70:1240-1246
68. Won J, de Evsikova CM, Smith RS, Hicks WL, Edwards MM, Longo-Guess C, Li T, Naggert JK, Nishina PM (2011) *NPHP4* is necessary for normal photoreceptor ribbon synapse maintenance and outer segment formation, and for sperm development. Human molecular genetics 20:482-496
69. Wang X, Zheng C, Liu W, Yang H (2017) Retinitis Pigmentosa and Bilateral Idiopathic Demyelinating Optic Neuritis in a 6-Year-Old Boy with OFD1 Gene Mutation. Case Rep Ophthalmol Med 2017:5310924
70. Jaeken J, Eggermont E, Stibler H (1987) An apparent homozygous X-linked disorder with carbohydrate-deficient serum glycoproteins. Lancet 2:1398
71. Roepman R, Bernoud-Hubac N, Schick DE, Maugeri A, Berger W, Ropers HH, Cremers FP, Ferreira PA (2000) The retinitis pigmentosa GTPase regulator (RPGR) interacts with novel transport-like proteins in the outer segments of rod photoreceptors. Hum Mol Genet 9:2095-2105
72. Khan AO, Al-Mesfer S, Al-Turkmani S, Bergmann C, Bolz HJ (2014) Genetic analysis of strictly defined Leber congenital amaurosis with (and without) neurodevelopmental delay. Br J Ophthalmol 98:1724-1728
73. Wolf M, Saunier S, O'Toole J, Wanner N, Groshong T, Attanasio M, Salomon R, Stallmach T, Sayer J, Waldherr R (2007) Mutational analysis of the *RPGRIP1L* gene in patients with Joubert syndrome and nephronophthisis. Kidney Int 72:1520-1526
74. Delous M, Baala L, Salomon R, Laclef C, Vierkotten J, Tory K, Golzio C, Lacoste T, Besse L, Ozilou C (2007) The ciliary gene *RPGRIP1L* is mutated in cerebello-oculo-renal syndrome (Joubert syndrome type B) and Meckel syndrome. Nat Genet 39:875-881
75. Billingsley G, Vincent A, Deveault C, Héon E (2012) Mutational analysis of *SDCCAG8 i*n Bardet-Biedl syndrome patients with renal involvement and absent polydactyly. Ophthalmic Genet 33:150-154.
76. Hull S, Kiray G, Chiang JP, Vincent AL (2020) Molecular and phenotypic investigation of a New Zealand cohort of childhood-onset retinal dystrophy. Am J Med Genet 184:708-717
77. Airik R, Slaats GG, Guo Z, Weiss AC, Khan N, Ghosh A, Hurd TW, Bekker-Jensen S, Schrøder JM, Elledge SJ, Andersen JS, Kispert A, Castelli M, Boletta A, Giles RH, Hildebrandt F (2014) Renal-retinal ciliopathy gene *Sdccag8* regulates DNA damage response signaling. J Am Soc Nephrol 25:2573-2583
78. Shaheen R, Faqeih E, Seidahmed MZ, Sunker A, Alali FE, AlQahtani K, Alkuraya FS (2011) A *TCTN2* mutation defines a novel Meckel Gruber syndrome locus. Hum Mutat 32:573-578
79. Huppke P, Wegener E, Böhrer-Rabel H, Bolz HJ, Zoll B, Gärtner J, Bergmann C (2015) Tectonic gene

mutations in patients with Joubert syndrome. Eur J Hum Genet 23:616-620
80. Sang L, Miller JJ, Corbit KC, Giles RH, Brauer MJ, Otto EA, Baye LM, Wen X, Scales SJ, Kwong M, Huntzicker EG, Sfakianos MK, Sandoval W, Bazan JF, Kulkarni P, Garcia-Gonzalo FR, Seol AD, O'Toole JF, Held S, Reutter HM, Lane WS, Rafiq MA, Noor A, Ansar M, Devi AR, Sheffield VC, Slusarski DC, Vincent JB, Doherty DA, Hildebrandt F, Reiter JF, Jackson PK (2011) Mapping the NPHP-JBTS-MKS protein network reveals ciliopathy disease genes and pathways. Cell 145:513-528
81. Thomas S, Legendre M, Saunier S, Bessières B, Alby C, Bonnière M, Toutain A, Loeuillet L, Szymanska K, Jossic F, Gaillard D, Yacoubi MT, Mougou-Zerelli S, David A, Barthez MA, Ville Y, Bole-Feysot C, Nitschke P, Lyonnet S, Munnich A, Johnson CA, Encha-Razavi F, Cormier-Daire V, Thauvin-Robinet C, Vekemans M, Attié-Bitach T (2012) *TCTN3* mutations cause Mohr-Majewski syndrome. Am J Hum Genet 91:372-378
82. Lee JH, Silhavy JL, Lee JE, Al-Gazali L, Thomas S, Davis EE, Bielas SL, Hill KJ, Iannicelli M, Brancati F, Gabriel SB, Russ C, Logan CV, Sharif SM, Bennett CP, Abe M, Hildebrandt F, Diplas BH, Attié-Bitach T, Katsanis N, Rajab A, Koul R, Sztriha L, Waters ER, Ferro-Novick S, Woods CG, Johnson CA, Valente EM, Zaki MS, Gleeson JG (2012) Evolutionarily assembled cis-regulatory module at a human ciliopathy locus. Science 335:966-969
83. Valente EM, Logan CV, Mougou-Zerelli S, Lee JH, Silhavy JL, Brancati F, Iannicelli M, Travaglini L, Romani S, Illi B, Adams M, Szymanska K, Mazzotta A, Lee JE, Tolentino JC, Swistun D, Salpietro CD, Fede C, Gabriel S, Russ C, Cibulskis K, Sougnez C, Hildebrandt F, Otto EA, Held S, Diplas BH, Davis EE, Mikula M, Strom CM, Ben-Zeev B, Lev D, Sagie TL, Michelson M, Yaron Y, Krause A, Boltshauser E, Elkhartoufi N, Roume J, Shalev S, Munnich A, Saunier S, Inglehearn C, Saad A, Alkindy A, Thomas S, Vekemans M, Dallapiccola B, Katsanis N, Johnson CA, Attié-Bitach T, Gleeson JG (2010) Mutations in *TMEM216* perturb ciliogenesis and cause Joubert, Meckel and related syndromes. Nat Genet 42:619-625
84. Srour M, Hamdan FF, Schwartzentruber JA, Patry L, Ospina LH, Shevell MI, Désilets V, Dobrzeniecka S, Mathonnet G, Lemyre E, Massicotte C, Labuda D, Amrom D, Andermann E, Sébire G, Maranda B, Rouleau GA, Majewski J, Michaud JL (2012) Mutations in *TMEM231* cause Joubert syndrome in French Canadians. J Med Genet 49:636-641
85. Maglic D, Stephen J, Malicdan MC, Guo J, Fischer R, Konzman D, Mullikin JC, Gahl WA, Vilboux T, Gunay-Aygun M (2016) *TMEM231* Gene Conversion Associated with Joubert and Meckel-Gruber Syndromes in the Same Family. Hum Mutat 37:1144-1148
86. Chih B, Liu P, Chinn Y, Chalouni C, Komuves LG, Hass PE, Sandoval W, Peterson AS (2011) A ciliopathy complex at the transition zone protects the cilia as a privileged membrane domain. Nat Cell Biol 14:61-72
87. Huang L, Szymanska K, Jensen VL, Janecke AR, Innes AM, Davis EE, Frosk P, Li C, Willer JR, Chodirker BN, Greenberg CR, McLeod DR, Bernier FP, Chudley AE, Müller T, Shboul M, Logan CV, Loucks CM, Beaulieu CL, Bowie RV, Bell SM, Adkins J, Zuniga FI, Ross KD, Wang J, Ban MR, Becker C, Nürnberg P, Douglas S, Craft CM, Akimenko M-A, Hegele RA, Ober C, Utermann G, Bolz HJ, Bulman DE, Katsanis N, Blacque OE, Doherty D, Parboosingh JS, Leroux MR, Johnson CA, Boycott KM (2011) *TMEM237* is mutated in individuals with a Joubert syndrome related disorder and expands the role of the TMEM family at the ciliary transition zone. Am J Hum Genet 89:713-730
88. Park E, Lee JM, Ahn YH, Kang HG, Ha, II, Lee JH, Park YS, Kim NK, Park WY, Cheong HI (2016) Hepatorenal fibrocystic diseases in children. Pediatr Nephrol 31:113-119
89. Otto EA, Tory K, Attanasio M, Zhou W, Chaki M, Paruchuri Y, Wise EL, Wolf MT, Utsch B, Becker C, Nürnberg G, Nürnberg P, Nayir A, Saunier S, Antignac C, Hildebrandt F (2009) Hypomorphic mutations in meckelin (*MKS3/TMEM67*) cause nephronophthisis with liver fibrosis (NPHP11). J Med Genet 46:663-670
90. Collin GB, Won J, Hicks WL, Cook SA, Nishina PM, Naggert JK (2012) Meckelin is necessary for photoreceptor intraciliary transport and outer segment morphogenesis. Invest Ophthalmol Vis Sci 53:967-974
91. Edwards M, Cha D, Krithika S, Johnson M, Parra EJ (2016) Analysis of iris surface features in

populations of diverse ancestry. R Soc Open Sci 3:150424
92. Chen L, Wei Y, Chi W, Fang D, Jiang X, Zhang S (2018) Potential Mutations in Chinese Pathologic Myopic Patients and Contributions to Phenotype. Curr Mol Med 18:689-697
93. Liu Q, Zhang Q, Pierce EA (2010) Photoreceptor sensory cilia and inherited retinal degeneration. Adv Exp Med Biol 664:223-232
94. Kim SK, Shindo A, Park TJ, Oh EC, Ghosh S, Gray RS, Lewis RA, Johnson CA, Attie-Bittach T, Katsanis N, Wallingford JB (2010) Planar cell polarity acts through septins to control collective cell movement and ciliogenesis. Science 329:1337-1340
95. Cui C, Chatterjee B, Lozito TP, Zhang Z, Francis RJ, Yagi H, Swanhart LM, Sanker S, Francis D, Yu Q, San Agustin JT, Puligilla C, Chatterjee T, Tansey T, Liu X, Kelley MW, Spiliotis ET, Kwiatkowski AV, Tuan R, Pazour GJ, Hukriede NA, Lo CW (2013) Wdpcp, a PCP protein required for ciliogenesis, regulates directional cell migration and cell polarity by direct modulation of the actin cytoskeleton. PLoS Biol 11:e1001720
96. Bredrup C, Saunier S, Oud MM, Fiskerstrand T, Hoischen A, Brackman D, Leh SM, Midtbø M, Filhol E, Bole-Feysot C, Nitschké P, Gilissen C, Haugen OH, Sanders J-SF, Stolte-Dijkstra I, Mans DA, Steenbergen EJ, Hamel BCJ, Matignon M, Pfundt R, Jeanpierre C, Boman H, Rødahl E, Veltman JA, Knappskog PM, Knoers NVAM, Roepman R, Arts HH (2011) Ciliopathies with skeletal anomalies and renal insufficiency due to mutations in the IFT-A gene *WDR19*. Am J Hum Genet 89:634-643
97. Coussa RG, Otto EA, Gee HY, Arthurs P, Ren H, Lopez I, Keser V, Fu Q, Faingold R, Khan A, Schwartzentruber J, Majewski J, Hildebrandt F, Koenekoop RK (2013) *WDR19*: an ancient, retrograde, intraflagellar ciliary protein is mutated in autosomal recessive retinitis pigmentosa and in Senior-Loken syndrome. Clin Genet 84:150-159
98. Fuster-García C, García-García G, Jaijo T, Blanco-Kelly F, Tian L, Hakonarson H, Ayuso C, Aller E, Millán JM (2019) Expanding the Genetic Landscape of Usher-Like Phenotypes. Invest Ophthalmol Vis Sci 60:4701-4710
99. Ashe A, Butterfield NC, Town L, Courtney AD, Cooper AN, Ferguson C, Barry R, Olsson F, Liem KF, Jr, Parton RG, Wainwright BJ, Anderson KV, Whitelaw E, Wicking C (2012) Mutations in mouse *Ift144* model the craniofacial, limb and rib defects in skeletal ciliopathies. Hum Mol Genet 21:1808-1823
100. Hoffer JL, Fryssira H, Konstantinidou AE, Ropers HH, Tzschach A (2013) Novel *WDR35* mutations in patients with cranioectodermal dysplasia (Sensenbrenner syndrome). Clin Genet 83:92-95
101. Gilissen C, Arts HH, Hoischen A, Spruijt L, Mans DA, Arts P, van Lier B, Steehouwer M, van Reeuwijk J, Kant SG, Roepman R, Knoers NV, Veltman JA, Brunner HG (2010) Exome sequencing identifies WDR35 variants involved in Sensenbrenner syndrome. Am J Hum Genet 87:418-423
102. Yamamura T, Morisada N, Nozu K, Minamikawa S, Ishimori S, Toyoshima D, Ninchoji T, Yasui M, Taniguchi-Ikeda M, Morioka I, Nakanishi K, Nishio H, Iijima K (2017) Rare renal ciliopathies in non-consanguineous families that were identified by targeted resequencing. Clin Exp Nephrol 21:136-142
